# Progress towards achieving child survival goals in Kenya after devolution: Geospatial analysis with scenario-based projections, 2015–2025

**DOI:** 10.1371/journal.pgph.0000686

**Published:** 2022-10-05

**Authors:** Noel K. Joseph, Peter M. Macharia, Emelda A. Okiro

**Affiliations:** 1 Population Health Unit, Kenya Medical Research Institute-Wellcome Trust Research Programme, Nairobi, Kenya; 2 Centre for Health Informatics, Computing, and Statistics, Lancaster Medical School, Lancaster University, Lancaster, United Kingdom; 3 Centre for Tropical Medicine and Global Health, Nuffield Department of Medicine, University of Oxford, Oxford, United Kingdom; United Nations, UNITED STATES

## Abstract

Subnational projections of under-5 mortality (U5M) have increasingly become an essential planning tool to support Sustainable Development Goals (SDGs) agenda and strategies for improving child survival. To support child health policy, planning, and tracking child development goals in Kenya, we projected U5M at units of health decision making. County-specific annual U5M were estimated using a multivariable Bayesian space-time hierarchical model based on intervention coverage from four alternate intervention scale-up scenarios assuming 1) the highest subnational intervention coverage in 2014, 2) projected coverage based on the fastest county-specific rate of change observed in the period between 2003–2014 for each intervention, 3) the projected national coverage based on 2003–2014 trends and 4) the country-specific targets of intervention coverage relative to business as usual (BAU) scenario. We compared the percentage change in U5M based on the four scale-up scenarios relative to BAU and examined the likelihood of reaching SDG 3.2 target of at least 25 deaths/1,000 livebirths by 2022 and 2025. Projections based on 10 factors assuming BAU, showed marginal reductions in U5M across counties with all the counties except Mandera county not achieving the SDG 3.2 target by 2025. Further, substantial reductions in U5M would be achieved based on the various intervention scale-up scenarios, with 63.8% (30), 74.5% (35), 46.8% (22) and 61.7% (29) counties achieving SDG target for scenarios 1,2,3 and 4 respectively by 2025. Scenario 2 yielded the highest reductions of U5M with individual scale-up of access to improved water, recommended treatment of fever and accelerated HIV prevalence reduction showing considerable impact on U5M reduction (≥ 20%) relative to BAU. Our results indicate that sustaining an ambitious intervention scale-up strategy matching the fastest rate observed between 2003–2014 would substantially reduce U5M in Kenya. However, despite this ambitious scale-up scenario, 25% (12 of 47) of the Kenya’s counties would still not achieve SDG 3.2 target by 2025.

## Background

Strategic decision-making processes are a critical element in health care and public health to inform the planning of future financial resources. A reliable health forecast is essential for health service delivery as it can enhance the delivery of health services and promote investments that make better use of limited sources. Therefore, tools to support stakeholders and policy makers in evidence-based decision-making have become increasingly important [1]. Various approaches are used in strategic decision-making in public health [2–5]. Scenario-based approaches [6] aim to connect a future scenario with the present while illustrating key decision points [1] and outline possible hypothetical non-arbitrary futures [7].

When combined with other sources of information, scenario-based modelling can improve and facilitate strategic and precise decision-making. In the last decade, integration of scenario modelling in forecasting tools such as the Lives Saved Tool (LiST), decomposition analysis, and the global burden disease (GBD) studies have been applied to identify knowledge gaps and propose new actions for reducing child mortality [8–10]. To improve child health outcomes and track progress towards targets during the Sustainable Development Goals (SDGs) era, such forecasting tools provide global, regional, and national trends that highlight how ongoing and future sustainable development efforts can be optimised. Additionally, reliable health forecasts that quantify inequities in child mortality within countries are prerequisites for planning equitable health interventions for the accelerated reduction in under-five mortality (U5M). However, such data are often not available or are aggregated at national which mask local level disparities [11].

There are few examples of scenario-based projections that evaluate progress in U5M reductions at the national level in Kenya. Keats and colleagues [12] identify high-impact interventions for more robust intervention programs that accelerate child mortality reductions. They predicted that the impact of scaling up of maternal, newborn and child health (MNCH) intervention packages to a coverage of 90% (an ideal scenario) would avert over 70% of under-five deaths by 2030. In a similar study, Hategeka et al. estimated that approximately 10,300 under-five deaths would be averted by scaling up ten community-level interventions to 99% by 2030 [13]. Although both studies indicate substantial declines in U5M by 2030, none have specifically examined the disparities on the potential impact of scaling up interventions and reducing disease infection prevalence at the units of health decision-making (counties) in Kenya. A subnational analysis is crucial to inform policies aimed at to eliminating health gaps in Kenya [14, 15]. Additionally, the ideal interventions scale-up scenarios evaluated are extremely ambitious and fail to account for local priorities or competing national priorities and resource constraints that determine intervention scale-up decision processes and implementation [16–18]. These knowledge gaps present the need for a more pragmatic description of the impact of scaling up interventions on U5M in Kenya informed by disaggregated local data and priorities grounded in actual policy-making processes outlined in the national or subnational strategic health plans rather than idealised targets.

This study uses country-specific information on U5M and interventions coverage to forecast subnational U5M rates based on recent available nationally representative health survey collected in 2014 in Kenya. Two pragmatic time-points of intervention coverage, 2022 (mid-term) and 2025 (end-term), were used to project U5M rates based on the four-intervention scale-up scenarios by assuming that all counties either achieve i) the highest subnational intervention coverage in 2014, ii) projected coverage based on the fastest county-specific rate of change observed in the period between 2003–2014 for each intervention, iii) the projected national coverage based on 2003–2014 trends and iv) the country-specific targets of intervention coverage. We then compare projected U5M with the current rates based on current trajectories of intervention coverage (business as usual). Finally, we track the achievement of SDG 3.2 across counties. This information will support subnational governments to better anticipate the future impact of current actions on specific outcomes and can influence long-term planning and investments.

## Methods

### Country context

Since the early 2000s, child mortality has declined remarkably in Kenya. Corresponding to the progress in child survival were continuous efforts to implement public health initiatives aligning with Millennium Development Goals (MDGs), that notably improved the coverage of maternal, newborn and child health (MNCH) intervention packages [12, 19–23]. However, significant challenges remain in attaining the SDG 3.2 target and the Kenya Vision 2030 long-term health objectives. This is compounded by underinvestment in health, underutilisation of health services and inequities in health service access within the country [21, 22, 24–26]. To effectively tackle challenges in attaining the SDG target, analyses focusing on drivers of inequities in child health are essential in understanding the contributions of competing risk factors and interventions to inform targeting and planning by decision-makers so that the gains made can be sustained and further accelerated. We have previously sought to address these gaps by assessing the contributions of 43 factors associated with U5M for the period between 1993 to 2014 sub-nationally in Kenya [26]. The analysis included a set of all potential predictors of U5M including those that are not directly amenable to intervention for a retrospective period. From the study, policy makers were able to understand the role played by different determinants in changes observed in child mortality during the MDG era [27]. However, here, we focus on identifying the most influential factors of U5M, amenable to interventions to inform targeted disease control, better resource allocation, focus on equity and maximising impact during the SDG era. This is important particularly to inform better planning by the policy makers targeting the most influential factors within defined projection scenarios that focus on accelerating declines in child mortality to meet both national (2022 and 2025) and global SDG targets (2025) on child survival. A summary of the differences in aims and methods of the current study and our previous study assessing the contributions of 43 factors associated with U5M are provided (Table A in S1 File).

In 2013, Kenya devolved health service delivery to 47 subnational units (counties) with the central objective of addressing inequities due to systematic disparities in access and healthcare service utilisation [28]. Accordingly, health service delivery is structured around the Kenya Essential Package for Health (KEPH) at four-level systems: community, primary health, country referral, and national referral services that underpin the SDGs principle of universal health coverage in Kenya [29]. County governments are responsible for providing services in three of the four levels, while the national government provides services at the national referral national services [30]. Recommendations from performance reviews of previous health outcomes and stakeholders evaluations used to guide health agendas across the country are operationalised at the national level according to the Kenya Health Sector Strategic plan (KHSSP) [31] or at the county level using the County Integrated Development Plans (CIDPs) [32]. Currently, both plans define an overall framework for mid-term financing and intervention coverage priorities for the period 2018–2023. To further accelerate the progress towards reduction of under-five mortality, these strategic plans recommend integrated approaches to address increased risk of child deaths among high-risk groups such as the immunocompromised. Therefore, we considered intervention coverage and disease-specific prevalence reduction among high-risk groups as priority programmes amenable to intervention. Forecasting is used to quantify the key results desired at the end of each period to demonstrate the outcomes and impacts expected from implementing the priority programmes outlined in the strategic plans based on previous trends and within budgetary constraints.

### Analysis overview

A subnational ecological analysis was carried out to project U5M rates under various scenarios of intervention scale-up after devolution in Kenya. First, a set of parsimonious factors significantly associated with U5M and amenable to interventions relevant to Kenya’s health priority programmes [31] were selected. Second, average county level annual rates of change (ARC) of U5M and the significant factors for the period between 2003 and 2014 were computed and used to estimate the continued trends from 2015 to 2025 (business as usual scenario-BAU). Third, a multivariable Bayesian space-time hierarchical model was used to predict country-specific annual counterfactual U5M assuming coverage from four alternate intervention scale-up scenarios, relative to BAU. Percentage change in U5M from the BAU coverage and counterfactual scenarios for each county and U5M estimates in 2022 and 2025 were reported. Finally, projected U5M rates for the years 2022 and 2025 were compared to the SDG 3.2 target of achieving <25 deaths per 1,000 live births for children below five years of age by 2030 to assess the potential impact on U5M reduction at county level in Kenya [33].

### Data

U5M rates at county level spanning years between 2003 and 2014 were available from previous work detailed elsewhere [34]. Briefly, the county specific U5M estimates were generated from birth histories for each household survey and census undertaken between 1989–2014 using five demographic methods. A bespoke Bayesian spatial-temporal Gaussian process regression model accounting for heterogeneity in demographic methods, sample size and household sample surveys was used to smooth the raw mortality estimates from demographic methods to predict county and national level U5M rates [34].

A broad range of factors known to influence U5M [12, 20, 35–37] including maternal interventions, pregnancy-related interventions, child health care seeking and preventative interventions, child health status, water, sanitation, and hygiene indicators (WASH) and community disease prevalence (Fig A in S1 File) available at the county level for the period 1993–2014 were considered for the analysis. Estimates of 43 factors were generated from household sample surveys and census conducted between 1993–2014 and smoothed using conditional autoregressive models as described elsewhere [22]. Of the 43 factors available, 29 factors (Fig A in S1 File) amenable to intervention were considered. Here, amenable to interventions refers to factors wholly or substantially adaptable to increased coverage or priority programmes aimed at reducing disease prevalence among high-risk groups (immunocompromised) grounded in Kenya’s health policy-making processes aimed at reducing U5M. Therefore, the prevalence of HIV and malnutrition indicators such as stunting were retained as a surrogate marker (risk factors) to track the impact of multiple concurrent interventions that often provide integrated approaches aimed at reducing the prevalence of HIV and stunting and their effect on U5M [38, 39]. Further HIV and malnutrition increase vulnerability to mortality due to immunocompromise which increases susceptibility to opportunistic diseases in addition to being health outcomes. We restricted our analysis to the baseline period between 2003–2014, coinciding with a period of intervention scale-up since most of the interventions such as bed nets, vitamin A and iron supplements were rolled out from the early 2000s [20, 22].

### Statistical analysis

#### Selection of interventions

Large-scale implementation of evidence-based interventions is as an effective tool to accelerate progress towards reduction of U5M that could prevent up to 70% of child deaths in low- and middle-income countries (LMICs) [15, 40]. Drawing from a previous study [22], we identified 27 factors amenable to intervention and disease prevalence reduction targeting high-risk groups (HIV and stunting) shown to have impact on U5M in Kenya and employed a multi-stage approach to select the most influential factors using previous trends. The focus of the study is to assess the impact on U5M after varied scale-up of interventions and reduction of the prevalence of HIV and stunting. The modelling approach assumes that changes in U5M are the result of changes in intervention coverage or reduction of disease prevalence and the impact of confounders such as distal factors (for example socioeconomic status) are mediated by changes in interventions coverage.

Bivariate associations between U5M and factors were assessed using a simple log-linear regression (Table B in S1 File). Among the covariates with significant effects (p<0.2), factors whose contribution was captured by other variables under consideration were excluded to reduce circularity and confounding [41]. For example, the effects of DTP3, Polio3, measles and BCG vaccines were captured by fully immunised status and were thus excluded in lieu of fully immunized indicator. Variables were further reduced through an elastic net regression (ENR) by selecting the most predictive factors via the *glmnet package* in R [42]. ENR shrinks the coefficients of redundant variables exactly to zero with the aim of selecting a part of the original variables to build a model, while selecting the most significant variables among highly correlated variables [43–45].

Two ENR models were used to account for antenatal visits (ANC), and interventions provided during ANC given their collinearity (Table C in S1 File). In addition to all the significant factors from the bivariate analysis, model 1 included ANC and each intervention offered during the visit. Model 2 was similar to model 1 except, it assessed the combined effect of interventions delivered during ANC as one index (Iron supplement, tetanus injection and Vitamin A supplements) derived from principal component analysis [46] (Table C in S1 File). Factors with non-zero coefficients from the ENR model with the least mean squared error were used in the subsequent models to assess the effect of interventions on U5M [44, 45].

#### Scenarios for projections

Four alternate intervention scale-up scenarios were used to project U5M estimates to 2022 and 2025. In the first time-point, interventions baseline coverage (2014) was scaled-up to match 2022 target values for the various scenarios while in the second time-point, estimates of intervention coverage attained in 2022 was scaled-up to the respective targets in 2025. These two time points reflect a pragmatic approach to intervention scale-up that constitutes mid-term and end-term goals aimed at achievable sustainable improvement of health outcomes proposed based on time-bound fiscal allocations in Kenya [28, 31, 32]. The potential impact of these four scenarios was measured against BAU estimates. To compute BAU; county-specific annual rates of change (ARC) for the period 2003–2014 were calculated and used to project U5M estimates to 2025 (S2 File). We, therefore, assumed that the trend for each intervention would remain constant over the study period and applied the ARC to the to the baseline estimates (2014) to get BAU trends for the period 2015–2022 and 2015–2025. The ARC is a constant rate of change between two time periods (2003 and 2014) expressed as a percentage (Eq 1)

$$ARC=\frac{\text{ln}[y_{ti+n}/y_{t}]}{n}*100$$ (1)

Where ***y***_***ti***_ is the U5M for a given county ***i*** in year ***t*** and ***n*** is the number of years between the two rates (e.g., 12 years when computing ARC from 2003 and 2014). The equation used to compute the ARC for each intervention coverage from 2022 or 2025 targets and the baseline coverage (2014 estimates) is provided in S1 File.

The four projection scenarios are described in Table 1 and the scenario target values used are presented in Table 2. Each scenario reflected a unique ambition of expanding the coverage of interventions based on the respective rationale yielding varied scale-up targets. This allowed for evaluation of the impact of different combinations of intervention coverage on U5M since each scenario represented varied intervention coverage targets with some scenarios having higher coverage of one intervention but lower coverage of another relative to the other scenarios. County-specific rates varied based on the corresponding baseline coverage (2014). For example, in scenario 1, if the coverage of health facility delivery for the best performing county in 2014 was 61.5%, all counties were matched to this coverage by 2022 using their respective coverage in 2014 as baseline values.

**Table 1:** Definitions, source of target values and rationale of intervention scale-up scenarios.

Scenario	Description	Projection strategy	Rationale
1	For each factor the coverage of the best performing county in 2014 was used as the target value for all counties in both 2022 and 2025 unless target coverage was achieved by 2022	Using the coverage of best performing county in 2014 and the 2014 national level estimates as baseline coverage, we computed a national annual rate of change (ARC) for each intervention. The computed national ARC was used to project intervention coverage to 2022 for each county. If a county did not attain the best performing county coverage by 2022, we maintained the computed ARC to project to 2025. For counties that reached the best performing county coverage estimates in 2022, we projected to a more ambitious universal coverage of 99% by 2025	A realistic target since intervention coverage of the best performing county in 2014 signify an optimal actual coverage achieved based on the existing data
2	The fastest county level ARC for each intervention (2003–2014) was used to project estimates for each intervention to 2022 and 2025	A constant annual rate of change (ARC) equal to the fastest ARC observed between 2003–2014 was used to project intervention coverage for the entire projection period (2015–2025).	Allows for evaluation of the impact of accelerated pace in coverage that coincides with a realistic measure of increased efforts to reduce child deaths over the last two decades in Kenya
3	The national ARC for each intervention (2003–2014) was used to project national coverage estimates to2022 and 2025 resulting in an average national target for each intervention	New ARCs for the periods 2015–22 and 2022–25 were computed using the computed national targets for 2022 and 2025 for each intervention. This ARC was then applied to each county to estimate county specific intervention coverage for each period. Because of different baseline values in each county, the projected coverage in both 2022 and 2025 differed by county.	An average rate of change meaning that no county is performing better than the other. Therefore, it reduces the gap between high and low performing counties to the country’s average reducing inequity
4	National targets for 2022 and 2025 from country’s strategic health plans including KHSSP 2018–2023 [31], the Kenya Malaria Strategy 2019–2023 [47] and WHO coverage guidelines [48]. Strategic health plan targets covered the period 2018–2023, therefore, 2023 target values were used as the scale-up target values in 2025.	Using 2022 and 2025 national intervention coverage targets defined in local or global health strategic plans, we computed a national ARC corresponding to the periods 2015–22 and 2022–25 separately. The computed national ARCs were then applied to 2014 county intervention estimates to project coverage for the periods 2015–2022 and 2022–2025. Similar to scenario 3, projected coverage in both 2022 and 2025 varied by county.	Incorporates previous coverage rates and the current financial constraints on health budgets pointing to anticipated coverage based on the specific country context and fiscal space

**Table 2:** National baseline coverage for interventions in 2014 and target coverage for the years 2022 and 2025, and the respective rate of change from baseline coverage for various intervention scale-up scenarios. Scenario 1; 2014 Best performing county coverage, scenario 2; fastest annual rate of change 2003–2014, scenario 3; 2022 and 2025 National projected coverage and scenario 4; 2022 and 2025 national health strategic plans targets.

Indicator	National Baseline coverage (%)	Scenario 1* 2022 and 2025 target coverage (annual rate %)	Scenario 2** 2022 and 2025 targets equal to the fastest annual rate % (2003–2014)	Scenario 3		Scenario 4***	
				2022 target coverage (annual rate %)	2025 targets coverage (annual rate%)	2022 target coverage (annual rate %)	2025 targets coverage (annual rate%)
ANC4	57.7	71.4 (3.0%)	5.3%	61.5 (0.8%)	62.9 (0.9%)	70 (2.7%)	80(4.8%)
Antimalarial use	33.5	51.9 (6.9%)	13.2%	40.1 (2.5%)	42.6 (2.7%)	70 (13.6%)	90(9.5%)
Breastfed within first one hour after birth	62.8	95.1 (6.4%)	11.2%	69.4 (1.3%)	72.1(1.5%)	70 (1.4%)	80 (4.8%)
Access to better sanitation	90.1	98.9 (1.2%)	13.0%	89.8 (-0.1%)	90.8(0.1%)	65 (-3.5%)	70 (2.5%)
Health facility delivery	61.5	89.2 (5.6%)	13.8%	76.5 (3.1%)	82.3(3.4%)	73 (2.3%)	75(2.2%)
Fever treatment	72.8	83.3 (1.8%)	4.3%	92.3 (3.4%)	99.6 (3.7%)	80 (1.2%)	90 (2.4%)
HIV	5.0	0.63 (-10.9%)	-9.1%	3.8 (-3.1%)	3.3 (-3.4%)	3.3 (-4.3%)	2.8 (-4.4%)
Child ITN use	56.0	76.8 (4.6%)	14.4%	83.5 (6.1%)	94.4 (6.9%)	80 (5.4%)	95 (7.0%)
Fully immunised	78.8	95.1 (2.8%)	9.8%	91.9 (2.1%)	96.9 (2.3%)	80 (0.2%)	85 (0.8%)
Access to better water	63.4	92.9 (5.8%)	7.8%	73.1 (1.9%)	76.8 (2.1%)	78 (2.3%)	80 (2.6%)

* The same annual rate of change was used for projections to 2025 where the target coverage was not achieved in 2022, otherwise it was projected to a universal coverage of 99% by 2025.

**The same annual rate of change was used for both 2022 and 2025 projections (ref to Table 1)

***Target values were sourced from [31, 47, 48].

#### Counterfactual analysis

A multivariable log-linear mixed effect spatio-temporal Bayesian ecological model was formulated to estimate the adjusted association of the final set of selected interventions on U5M using WinBUGS Package (Version 1.4.3) [49]. The model included an intercept, fixed effects, spatial and temporal random effects, and a space-time interaction effect. The random effects were assumed to follow prior distributions that capture the spatial-temporal structure of U5M by borrowing information across space and time and time-varying effects that influence all counties (unstructured) and those that are county-specific (structured). Assumptions about intervention coverage and how their associations with U5M were modelled are provided in S1 File and the details of full specification of the model can be found in [26]. Coefficients of the full multivariable model were fit using a two-chain Markov chain Monte Carlo (MCMC) simulation to improve the precision of the parameter estimates and their respective posterior distributions of parameters summarized by the mean and the 95% credible intervals (CI). A total of 25,000 iterations were run per chain including a burn-in period of 5,000 per chain to generate acceptable Monte Carlo errors (<5% of the posterior standard deviation). Trace plots and Gelman-Rubin statistic were used to assess model convergence towards the target distribution of the parameters while Monte Carlo error, the standard deviation and their ratio were used to evaluate model accuracy (the level of uncertainty of the estimated parameters) [50, 51]. The additional value of using both the trace plots and Gelman’s statistic to assess convergence, is that Gelman’s statistic compares the spreads of the individual chains with the spread of all the draws pooled to inform how close the chains converge on parameters target distribution. We adapted a Gelman statistic threshold of less than 5% that has previously been shown to infer to the model stability [50].

The adjusted coefficients were used to predict county-specific annual counterfactual U5M estimates assuming intervention coverage from the four scale-up scenarios relative to coverage under BAU. The percentage change in U5M from the BAU and all four scale-up scenarios were computed for each county to quantify the impact of all selected interventions jointly as well as for each intervention for the period 2015–2025. Finally, U5M estimates for the year 2022 and 2025 from each scale-up scenario and the BAU coverage were compared to the SDG 3.2 target of achieving <25 deaths per 1,000 live births for children below five years of age to evaluate progress in 2022 and 2025. Data preparation and pre-processing were done in StataCorp. 2014 [Stata Statistical Software: Release 14. College Station, TX: StataCorp LP]. ArcMap 10.5 (ESRI Inc., Redlands, CA, USA) was used to create maps for visualization of the projected U5M estimates in 2022 and 2025.

#### Ethics approval and consent to participate

The manuscript does not contain any individual level data.

## Results

Eleven parsimonious factors and amenable to intervention were retained from the modelling building process and included in the Bayesian ecological space–time mixed-effects regression; the details are presented in (Fig A in S1 File). The 11 factors included four antenatal visits, antimalarial use, breastfeeding within first hour of birth, access to better sanitation and water for drinking, health facility delivery, seeking treatment for fever, HIV prevalence, child ITN use, stunting and fully immunised status. Stunting was ultimately not statistically significant and hence was excluded from the final model used for counterfactual analysis. Table 3 shows the results from the Bayesian ecological space–time mixed-effects regression.

**Table 3:** The mean regression coefficients, 2.5–97.5% quantiles effects, SD, MC error and ratio of 10 interventions considered for scale up scenarios using the ecological Bayesian spatio-temporal mixed-effect regression model.

Indicator	Coefficient (95%CI)	SD	MC error	Gelman statistic (%)
ANC4	-0.7098 (-0.7063–-0.5112)	0.1039	0.004037	3.8855
Antimalarial use	-0.0416 (-0.1569–-0.0135)	0.05919	0.002032	3.4331
Breastfed within first hour after birth	-0.4162 (-0.5557–0.2816)	0.06921	0.003059	4.4199
Access to better sanitation	-0.2136 (-0.3166–0.1089)	0.06401	0.002702	4.2212
Health facility delivery	-0.4740 (-0.6457–0.3019)	0.09827	0.004554	4.6342
Fever treatment	-1.2550 (-1.451–1.056)	0.1006	0.003319	3.2992
HIV	6.5930 (4.962–8.8188)	0.9968	0.04802	4.8174
Child ITN use	-0.0065 (-0.0828–0.0064)	0.03818	0.0014	3.6668
Fully immunised	-0.5817 (-0.7378–0.4232)	0.08059	0.003189	3.9571
Access to better water	-0.3558 (-0.5259–0.1853)	0.08707	0.003864	4.4378
Intercept	-0.9273 (-1.136–0.724)	0.1047	0.005052	4.8252
sigma.nu [1]	0.0024 (8.108E-5-0.009975)	0.004936	1.90E-04	3.8391
sigma.nu [2]	0.1398 (0.09825–0.1962)	0.02519	0.001008	4.0016
sigma.t	0.0591 (0.03867–0.0926)	0.01387	2.11E-04	1.5227
sigma. w	0.5336 (0.4315–0.6643)	0.05943	0.001155	1.9435

Increasing intervention coverage and reduced disease prevalence were associated with varied impacts on U5M rates (Table 3). The coefficients represent the effect of a factor equivalent to a unit change (percentage) on under-five mortality adjusting for all the other predictors included in the model. HIV prevalence was associated with the largest increase in U5M of 6.5930 (95% CI: 4.962–8.8188) while seeking treatment after fever; -1.2550 (95% CI: -1.451–-1.056) was associated with the greatest decline in U5M relative to the other factors (Table 3). All the model parameters in each successive iteration of MCMC sampling chains converged as illustrated by the trace plots (S3 File) as well as the Gelman statistic of <5% shown in Table 3. Estimates of the coefficients were within the acceptable range of uncertainty with MC errors<1.5% for all predictors. The random effects parameters indicate a relatively higher spatial variation (*sigma*.*w*) compared to temporal variation (*sigma*.*t*) in our analysis (Table 3).

### Projection rates

Nationally, child deaths declined from 69.8 (95% CI; 59.2–80.2) per 1,000 livebirths in 2003 to 59.5 (95% CI; 53.1–65.8) per 1,000 livebirths in 2014, translating to an absolute decline 17.3% and ARC of -1.4% (95% CI: -1.61–0.54). County-specific annualised rates of U5M change were varied ranging from -4.3% in Mandera county to 2.9% in Nyandarua county with 28% (13 of 47) of the counties witnessing an increasing U5M ARC (S3 File). Child ITN use had the highest mean ARC of 22.1% (95% CI: 21.0–23.2). HIV prevalence had a decreasing rate of change of -4.71 (95% CI: -5.17–4.25), while health facility delivery and fully immunised had an increasing rate of change of 4.9% (95% CI:4.2–5.6) and 3.5% (95% CI: 2.9–4.0), respectfully. All the other interventions had an increasing mean rate of change of 2.5%.

### Counterfactual U5M

**Fig 1:**
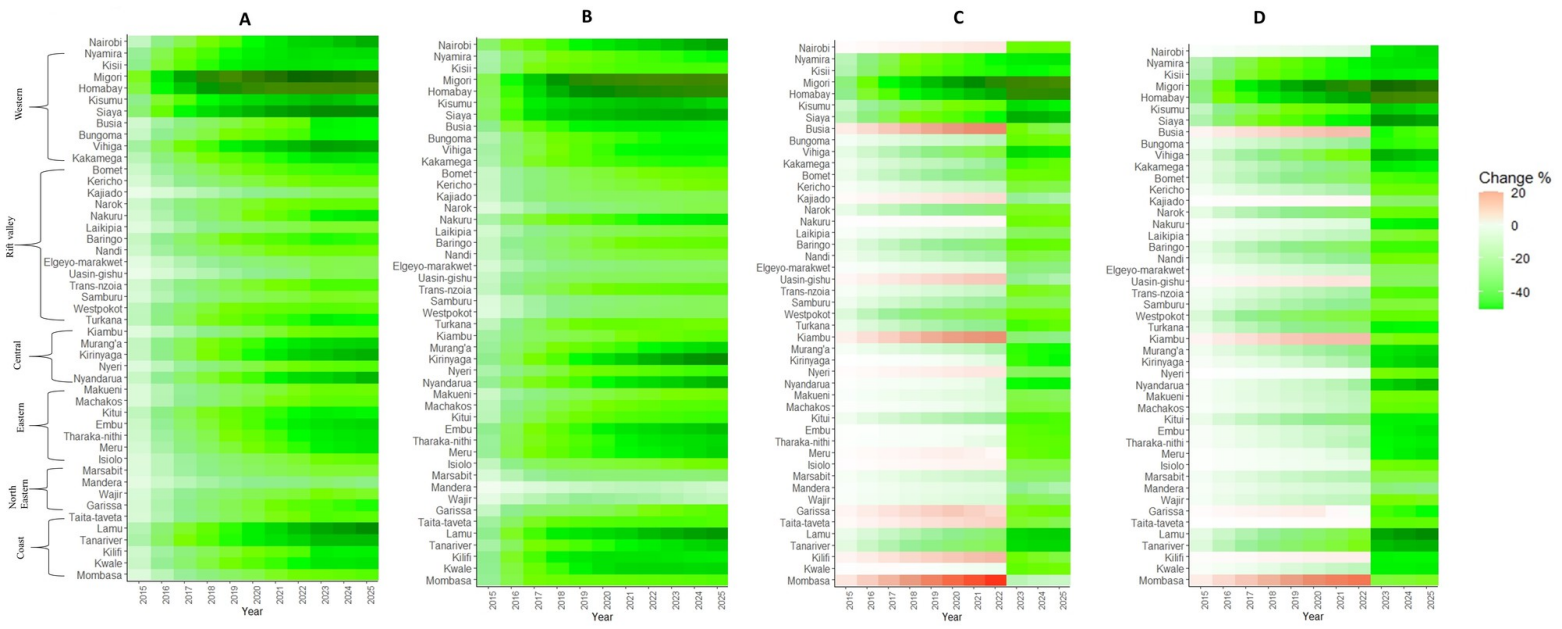
Percentage change in U5M relative to 2014 after scale-up of all 10 interventions using different coverage scenarios relative to BAU for 2022 and 2025. A) Scenario 1; 2014 Best performing county coverage, B) Scenario 2; Fastest annual rate of change 2003–2014 C) Scenario 3; 2022 and 2025 National projected coverage and D) Scenario 4; 2022 and 2025 national health strategic plans targets.

Wide disparities in U5M reduction exist across counties based on the different scale up scenarios relative to the BAU coverage (Fig 1). Scenario 1 scale-up targets had the greatest impact in U5M across all the counties in 2022 while scenario 2 had the greatest impact in 2025. For scenario 1, the mean reduction was -28.4% (95%CI: -31.8–-24.9) in 2022 ranging from -69.1% in Migori county to -110% in Mandera county relative to BAU. By 2025, the mean reduction in U5M improved to -31.3% (95%CI: -34.7–-27.8) relative to BAU. Thirty-eight percent (18 of 47 counties) of the counties would achieve reductions of at least 30% in 2022 with an additional 13% (6 counties) when the timeline is extended to 2025 (Fig 1A).

Scale-up using scenario 2 would achieve relatively similar reductions compared to scenario 1 of -26.5% (95%CI: -29.8–-23.2) in 2022 with small improvements to -33.1% (95%CI: -36.9–-29.4) in 2025 relative to BAU (Fig 1B). Notably, counties characterized by low baseline intervention coverage (marginalized) such as Wajir, Mandera, Garissa and Marsabit attain greater reductions in U5M using scenario 1 scale-up while scenario 2 would produce increased reductions for counties with relatively higher intervention coverage such as Nairobi, Kirinyaga and Nyandarua (Fig 1A and 1B).

Scale-up of interventions under scenario 3 using projected national coverage to 2022 would attain smaller impact on U5M (relative to scenario 1 and 2) with a mean reduction of -7.6% (95% CI: -11.6–-3.7) in 2022 and substantially greater impact of -22.8% (95% CI:-26.1–-19.5) by 2025. Based on this scenario, in 2022, 30% (14 of 47) of the counties would have higher U5M rates relative to BAU. However, all these counties would achieve lower U5M rates in 2025 compared to BAU (Fig 1C). This reversal observed in trends in U5M rates from 2022 to 2025 was mainly associated to scale-up of five interventions corresponding to mean changes in U5M rates as follows: four antenatal care visits (-22%), health facility delivery (-19.4%), breastfeeding within first hour after birth (-19.3%), HIV prevalence (-16%) and fully immunised (-7.1%) (S4 File).

Scenario 4 had an average change in U5M rates of -10.8% (95% CI: -14.6–-7.0) and -29.9% (95% CI: -33.2–-26.6) relative to BAU coverage by 2022 and 2025, respectively. Fifteen percent (7 of 47 counties) had worse U5M rates relative to BAU coverage ranging from 13.5% in Mombasa to 0.4% in Taita Taveta in 2022. Individual intervention scale-up to 2025 target values reflects further improvements in U5M rates in 2025 for majority of interventions except health facility delivery, Child ITN use and access to better sanitation in the seven counties relative to BAU (S4 File).

There were key differences across counties and scale up scenarios with respect to impact of individual interventions on U5M rates in 2025 (S4 File). Consequently, for optimal reduction in U5M, different combinations of targets values across scale scenarios can be derived. For example, scale-up of health facility deliveries, early breastfeeding and antimalarial use to scenario 1 targets produced the highest reductions in U5M rates compared to all the other scenarios. Further, intervening on improved access to safe water for drinking, antenatal care visits, full immunisation coverage, and reduction in HIV prevalence based on scenario 2 targets, and improvement in seeking treatment after fever based on scenario 3 targets, in combination would yield the most reductions in U5M rates. By 2025, projected U5M rates for BAU based on access to improved sanitation and child ITN use was similar to projected U5M rates from all the scale-up scenarios. In summary, reducing HIV prevalence (scenario 2), scale-up of access to improved water (scenario 2) and recommended treatment of fever (based on scenario 3) yield the highest reduction in U5M by 2025. The corresponding reduction in U5M would be 28.2% (95% CI: -55.2–-1.1) and -22.7% (95% CI: -44.5–-1.0) and -19.2% (95% CI: -37.6–-0.9), respectively.

### Projected U5M estimates and SDG targets

**Fig 2:**
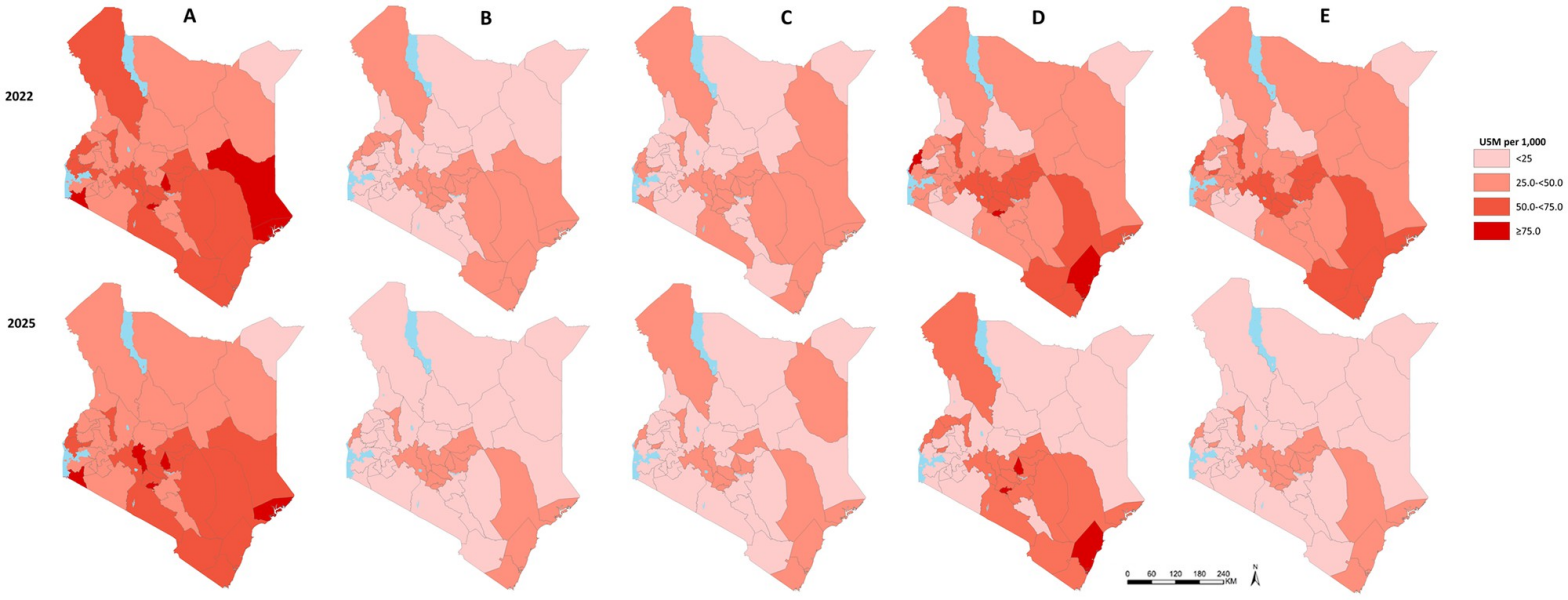
Projected U5M rates in 2022 and 2025 using scale up of 10 interventions different scenarios. A) Business as usual, B) Scenario 1; 2014 Best performing county coverage, C) Scenario 2; Fastest rate of change of factors between 2003–2014 D) Scenario 3; National projected coverage and E) Scenario 4; national health strategic plans targets. Source: The Kenya county level boundaries outline shapefile was obtained from [52].

Fig 2 presents U5M estimates in 2022 and 2025 under the various scenarios. Collective scale-up of all nine interventions and reduction of HIV prevalence to targets across all scenarios would attain lower U5M rates compared to BAU. The country’s U5M estimates under BAU would be 53.3 (95% CI: 48.4–58.1) by 2022 and 51.0 (95% CI: 46.1–56.0) per 1,000 livebirths by 2025. In 2022, scenario 1 would have the least U5M rates of 25.3 (95% CI: 22.2–28.4) per 1,000 livebirths while scenarios 2, 3 and 4 would have been 27.1 (95% CI: 24.6–29.7), 46.0 (95% CI: 40.3–51.7) and 42.8 (95% CI: 37.5–48.1) child deaths per 1,000 livebirths, respectively. In 2025, scenario 2 scale-up targets yield better U5M rates of 18.3 (95% CI: 16.0–20.6) deaths per 1,000 livebirths, closely followed by scenario 1 with 20.2 (95% CI: 17.2–23.2) and scenario 4 that would attain 21.6 (95% CI: 18.3–27.8) per 1,000 live births. Scenario 3 scale-up yielded the highest U5M rates of 28.7 (95% CI: 24.6–32.7).

Only one county (Mandera) would achieve the SDG target by 2025 while 30% (14) of counties would have U5M rate >50 per 1,000 livebirths when maintaining business as usual coverage. Scale-up of interventions to scenario 1 (best performing county values), 2025 national projected values (scenario 3) and scenario 4 (the national strategic plans targets) would increase the number of counties achieving the SDG target to 61.7% (29 counties), 46.8% (22 counties) and 63.8% (30 counties) respectively. More ambitiously, if every county would manage to scale up interventions coverage to scenario 2 targets (fastest rate of change between 2003–2014), 74.5% (35 of 47 counties) would achieve SDG U5M rates target by 2025. Notably, counties that did not achieve U5M rate of at least 25 per 1,000 livebirths in 2025 after scenario 2 scale-up had an increasing ARC between 2003 and 2014 or U5M rate of >90 per 1,000 livebirths in 2014. Despite failure to attain the SDG target, U5M rates for these counties were significantly lower compared to all other scale-up scenarios ranging from 31.6 to 26.0 child deaths per 1,000 livebirths in Nyandarua and Murang’a counties respectively (Fig 2).

## Discussion

Our study extends previous efforts that provide evidence on progress towards improving child survival and attainment of child sustainable development goals in Kenya [12, 13], by evaluating the potential impact of scaling up multiple child health interventions on U5M reduction, for which sufficient evidence at county level is lacking. We projected U5M rates at county level based on historical trends for the period 2003 to 2014 (BAU) and illustrated the potential benefits of four alternative scale up coverage scenarios (Table 1) for the period between 2015–2025 to assess relative progress towards SDG targets of reducing U5M to at least 25 deaths per 1,000 live births. Our findings indicate that without any acceleration in the pace of coverage for the selected interventions (BAU), the average U5M rate would be 51.0 (95% CI: 46.1–56.0) deaths per 1,000 livebirths. All the counties except Mandera county would not achieve the SDG target by 2025. The alternative projection scenarios show substantial advances towards achieving SDG target, with scenario 2 (scale-up corresponding to the fastest rate of change in intervention coverage achieved between 2003–2014) resulting to the highest reductions in U5M rates relative to BAU (Fig 1). Overall, 63.8% (30), 74.5% (35), 46.8% (22) and 61.7% (29) counties achieve SDG target for scenario 1 (best performing county coverage), scenario 2 (fastest rate of change), scenario 3 (the national projected value) and scenario 4 (the Kenya national health strategic plans targets) respectively by 2025 (Fig 2).

Projections based on BAU, highlight the enormity of the challenge of achieving the SDG aspiration based on pre-existing trends of intervention coverage as counties are making slow progress in reducing U5M (Fig 2A). Notably, fifty-one percent of the counties (24 of 47) located in central and coastal regions of the country were projected to have U5M rates >50 deaths per 1,000 livebirths in 2025 based on BAU. These results correspond to the findings from a recent study by Wakefield et al, who estimated a relatively slow decline in U5M across counties in 2020 and that continuing past trends in annual rates of U5M reduction would not be sufficient to reach the SDG target for most counties. In addition, counties within the central, western and coastal regions were projected to have the highest burden of U5M [53]. Further, the UN Inter-agency Group for Child Mortality Estimation estimated the average U5M rates in Kenya to be 42.3 (95% CI: 33–55) deaths per 1,000 livebirths in 2019 indicating potential for missed SDG target by 2030 [54–58], similar to our current findings.

These findings highlight the need for evidence-based strategies to inform subnational efforts aimed at accelerating the pace of U5M decline to increase the likelihood of achieving SDGs. However, formulation of such strategies is challenged by insufficient evidence on the implications of intervention scale-up due to scarcity of data. Sample household surveys conducted every 3 to 5 years have been the main data sources and are often not powered to generate U5M estimates at lower units used for decision making. Long-term improved monitoring of U5M requires strengthening of data collection from routine health system information such as the District Health Information System (DHIS) and health demographic surveillance systems (HDSS) to gather timely, disaggregated data that can inform evidence-based decision in Kenya [55–58]. Therefore, due to lack of recent data from sample surveys or strengthened HMIS, projections based on BAU may underestimate the magnitude of U5M reduction especially for counties that have recently intensified interventions and investments to improve child health.

In our analysis, scenario 2 yielded the highest reductions in U5M among all intervention coverage scale up scenarios, with an overall projection of 18.3(95% CI: 16.0–20.6) deaths per 1,000 livebirths translating to an absolute decline of 64.7% relative to BAU by 2025. This scenario illustrates the potential benefits of a rigorous scale up approach that maintains a long-term focus for optimal coverage achievable while the other scenarios depict an opportunity for further improvements beyond the targets set. These projections should be interpreted with caution since we assume that the fastest rate of change (2003–2014) would be maintained to 2025, however, in reality, the socio-economic and demographic environment that children are born and raised is not a smooth occurrence and can create unpredictable mortality shocks [22, 59, 60]. Additionally, the model does not capture the effects of health delivery systems that influence the quality of interventions delivered and utilisation that are crucial in reducing U5M [56]. Future analysis to evaluate the validity of the impact of intervention scale-up scenarios is necessary. For example, the 2019 national census data when available can be used to validate the scenarios and create a baseline for future analyses.

Strategizing for health at subnational levels as well as continued context-specific prioritisation of key drivers of U5M reduction is crucial in reducing U5M inequities within the country. Differences on the impact of individual or a combination of the 10 factors considered in the analysis on county-level U5M rates based on the four scale up scenarios were varied (Fig 1 and S4 File). Overall, accelerated pace of HIV prevalence reduction, scale-up of access to improved water and recommended treatment of fever would have considerable impact on U5M reduction (≥ 20%) using scenario 2 coverage (S4 File). Forecasted estimates of U5M rates for counties in northeastern, eastern, and northern Rift Valley regions with worsening, or consistently low coverage based on the pre-existing trends relative to other areas of the country showed steady improvements from 2015 to 2025, indicating substantial U5M reductions for all intervention coverage scale-up (Fig 1). Similarly, findings from other studies have reported that counties with relatively low coverage of interventions would have substantial gains in reductions of U5M after deliberate prioritization of vulnerable subpopulations often characterized by low intervention coverage and utilization [13, 20, 61]. This is line with SDG’s overarching goal of reaching the most marginalized, first. Conversely, counties with high intervention coverage such as Kirinyaga, Kiambu, Nyeri and Nairobi in 2014 require an ambitious scale-up approach such as scenario 2 with consistent improvement that aim towards universal coverage for substantial progress towards U5M reduction. These findings illustrate the potential benefits of a general mixed approach to guide targeting of intervention scale-up by characterizing different counties based on pre-existing trends in intervention coverage to ensure sustainable and equitable progress towards U5M reduction in Kenya. For example, setting county-specific targets aimed at steady improvements in coverage of high-impact interventions for counties deemed to have high coverage in 2014 as illustrated in scenario 2 while trying to improve poor performing counties to catch-up with best performing counties coverage in 2014 as illustrated in scenario 1.

Aspects of our analysis reflect a comprehensive approach comparable to intervention scale-up strategies adopted within the Kenyan context based on time-bound fiscal budgets [28, 31, 32]. Intervention scale-up scenarios incorporated mid-term and long-term targets to assess the impact of time-bound implementation plans on U5M reduction. In our analysis, we note substantial shifts in U5M reduction based on 2022 (short-term targets) and 2025 (long-term targets) (Fig 1). For example, scenario 4 that is informed by local health strategies shows accelerated U5M reduction post-2022 (Fig 1D) implying that the short-term intervention targets would yield marginal reductions in U5M rates for majority of the counties relative to BAU. Gaps between the 2022 and 2025 scale-up scenarios provide quantification of the impact of short-term priorities on future health trajectories indicating looming stagnation, reversal or accelerated pace for progress. Therefore, provisions that allow evaluation and re-adjustment of targets relative to evidence-based trends are integral part of the development process of a robust policy making.

Overall, findings from this study demonstrate that Kenya has opportunities to further reduce U5M and that varied scale-up scenarios are better positioned for improvement than others. Currently, the CIDPs provide targets to aid local level planning and budgeting to ensure that basic human development goals including child survival are responsive to long term national goals such as the Kenya Vision 2030 and the SDGs. The CIDPs focus on cross cutting issues affecting development in the county for resource mobilisation [32] which requires evaluations to determine the collective effects achieved by scaling up different interventions on specific health outcomes such as U5M. In the past decade, there has been substantial investments in the development of standardised methods such as LiST and *spectrum* that illustrate scenario projections aimed at assessing the impact of intervention scale-up on various health outcomes such as child mortality. These methods, however, focus on biomedical interventions used to evaluate disease-specific cause of death and often do not consider the effect of broader social determinants of health [62–64]. By contrast, in this study, we propose hypothetical scale-up scenarios based on historical trends as well as intervention coverage scale-up proposed by health stakeholders in the country for cost-effective interventions taking into account broad social determinants such as improved water and sanitation to compute future trends. Integration of comprehensive evidence-based projections such as those presented here, are a starting point to aid better decision making at county units when planning policy for upcoming years and for deciding between investment possibilities to optimize resource utilization.

### Limitations

There were several study limitations. First, there were no available county specific U5M rates and intervention coverage data for the period between 2015–2020. Hence, data used for the baseline year (2015) was model-based and subject to uncertainty [34]. Our modelling exercise assumes that historical trends in associations between interventions and U5M from the observed data (2003–2014) would still apply over the study period between 2015–2025 (projection period). Given the lack of current nationally representative data on U5M and factors of U5M, we kept coefficients generated using 2003–2014 data as it was impossible to rigorously assess the current associations post-2014 in Kenya. It is possible that, the true decline of U5M rates for the period between 2015–2025 might be greater than we estimate for counties that recently intensified interventions and investments to reduce their U5M rates post 2014. However, we developed scenario-based projections reflecting a range of reasonable values from the recently available data sources to capture empirical data-driven county-specific trends (Table 2). In addition, disruptions in the provision and utilisation of routine health services and the broader socioeconomic effects of the COVID-19 pandemic threaten countries’ ability to achieve SDG targets [65]. Recent estimates suggest a potential increase in child mortality up to an additional 1·2 million child deaths in low-income and middle-income countries as a result of essential health services utilisation drops associated to COVID-19 [36]. In our analysis, the subset of interventions identified as the most impactful in reduction of U5M are based on pre-COVID-19 estimates of U5M trends and baseline intervention coverages. Large and disproportionate changes to provision and utilisation of the identified interventions or prevention and management of HIV/AIDS and malaria may imply changes in the prioritisation and scale up of interventions. Further work is required to investigate the acute impacts of COVID-19 in prioritisation and scale-up of interventions with regards to U5M reductions.

Second, there are potentially strong assumptions made about interventions coverage and how their associations with U5M were modelled (S1 File). We employed linear interpolation to estimate the impact of various interventions. This modelling process inherently, assumes that the impact of increasing intervention coverage or reduction of disease prevalence would be equivalent regardless of the initial values. For example, the impact of increasing coverage of antenatal care visits from 80% to 90% coverage would be equivalent to that of increasing coverage from 10% to 20%. It is likely that the scale-up level would not follow the linear increment assumed in the model and that the efforts required to scale up interventions from very low values would be similar when the existing coverage is deemed high such as >80%. We also assume that intervention scale-up independently and additively affect U5M. However, we cannot rule out that depending on the nature of the interaction between interventions, scale-up of a particular intervention might have influence in coverage levels of another. For example, increased coverage of access to clean water might lead to improved access to sanitation. These limitations suggest that future research should focus on improving both data availability and the integration in scenario-based models.

Further, the interventions reviewed were chosen for programmatic reasons. Although the interventions studied were strongly associated and of high impact in reduction of U5M, inclusion of long-term impact interventions such as maternal education and health systems indicators such as quality of care would improve child mortality estimation to monitor progress in U5M reduction. We interpret these results with caution as evidence of partial causal effects of the selected child survival interventions assuming that there are no omitted effective interventions in this analysis. We were unable to include data from all the sources available such as routine data when computing the parameter estimates, as only average values from household surveys at county-units were available. This limitation is likely to have a non-systematic impact on the results and projections since earlier studies have established the validity and reliability of survey estimates [66]. Finally, we considered HIV and stunting as risk factors that increase vulnerability to U5M due to immunocompromise which increases susceptibility to opportunistic diseases. However, an alternative model formulation could consider integrated packages that directly or indirectly reduce the burden of HIV and stunting on U5M when such data is available. For example, HIV counselling and testing, prevention of mother-to-child transmission (PMTCT) and prompt treatment of HIV opportunistic infections.

## Conclusion

This analysis gives an account of the potential impact on U5M reduction resulting from acceleration programs illustrated using four hypothetical intervention scale-up scenarios at subnational units in Kenya. Of the ten factors significantly associated to U5M, emphasis on accelerated pace of HIV prevalence reduction, scale-up of access to improved water and recommended treatment of fever would have considerable impact on U5M reduction. Without intensified efforts to scale up interventions (BAU), counties would have marginal reductions in U5M. Contrary, substantial reductions in U5M would be attained based on the scale up scenarios evaluated with the number of counties achieving the SDG 3.2 target by 2025 ranging from 35 (74.5%) to 22 (46.8%). Concerted efforts and resource allocation by the local government as well as other key health stakeholders to accelerate the pace of progress to fulfil children’s rights to health and development are critical. Such efforts need to be supported by well-designed and robust scale-up strategies to effectively reach sub-groups of the population such as the vulnerable populations characterised by poor intervention coverage and utilization as well as promote universal coverage for substantial progress towards achieving the child health related SDG target in Kenya.

## Supporting information

S1 File Factor selection processes. Table A: a summary of the differences in aims and methods of the current study and our previous study assessing the contribution of factors associated to U5M [26], Fig A: Schematic strategy showing factor selection process, Table B: Determinants of child mortality amen*a*ble to interventions and crude association with *under-five mortality* for the period 2003–2014 and Table C: Alternate Elastic Net Regression results for variable selection.
(DOCX)

S2 File County-specific annualised rates of change (ARC) for the period between 2003–2014. (DOCX)

S3 File Trace plot history for model parameter convergence assessment. (DOCX)

S4 File Percentage changes in U5M rates after scaling up of individual selected factors associated with U5M for each intervention scale-up scenario. (PPTX)
