# Supplementary material for: Progress towards achieving child survival goals in Kenya after devolution: Geospatial analysis with scenario-based projections, 2015–2025
Source: PLOS Glob Public Health. 2022 Oct 5;2(10):e0000686. doi: 10.1371/journal.pgph.0000686 (PMC10021401; doi:10.1371/journal.pgph.0000686)
Supplement: S1 File — Table A: a summary of the differences in aims and methods of the current study and our previous study assessing the contribution of factors associated to U5M [26], Fig A: Schematic strategy showing factor selection process, Table B: Determinants of child mortality amenable to interventions and crude association with under-five mortality for the period 2003–2014 and Table C: Alternate Elastic Net Regression results for variable selection. (DOCX) [file pgph.0000686.s001.docx]

Table A: A summary of the differences between the aims and the methods in the current and previous study assessing the contribution of factors of U5M [1]

| Macharia et al. (2021) | Current study |
| --- | --- |
| Aim: Identifying the most influential factors of U5M, amenable to interventions to inform targeted disease control, better resource allocation, focus on equity and maximising impact during the SDG era | Aim: To assess the role played by different determinants in changes observed in child mortality during the MDG era |
| Retrospective study (1993-2014) | Prospective study with forecasts for the time period 2015-2025 based on previous trends (2003-2014) |
| Considered 43 factors not necessarily amenable to intervention | Considered 27 of 43 factors amenable to intervention and disease prevalence reduction among high-risk target groups (HIV and stunting) as proxy for multiple interventions |
| Elastic net regression (ENR) was used to reduce the set of all significant factors (24 of 43 factors) where factors with non-zero coefficients formed the base model | ENR was used to select the most predictive factors to be included in the final model while accounting for multicollinearity between ANC visits and interventions delivered during ANC |
| Further simplification of the base model from ENR was explored by assessing DIC of alternate models fit based on different combinations of predictive factors | ENR model with the least MSE was adopted for counterfactual analysis without further simplification |
| Counterfactual analysis quantifies the impact of coverage of factors of U5M on child mortality based on observed data | Counterfactual analysis quantifies the impact of hypothetical scale-up scenarios on U5M |

**Fig A****: Schematic strategy showing** **factor selection process**
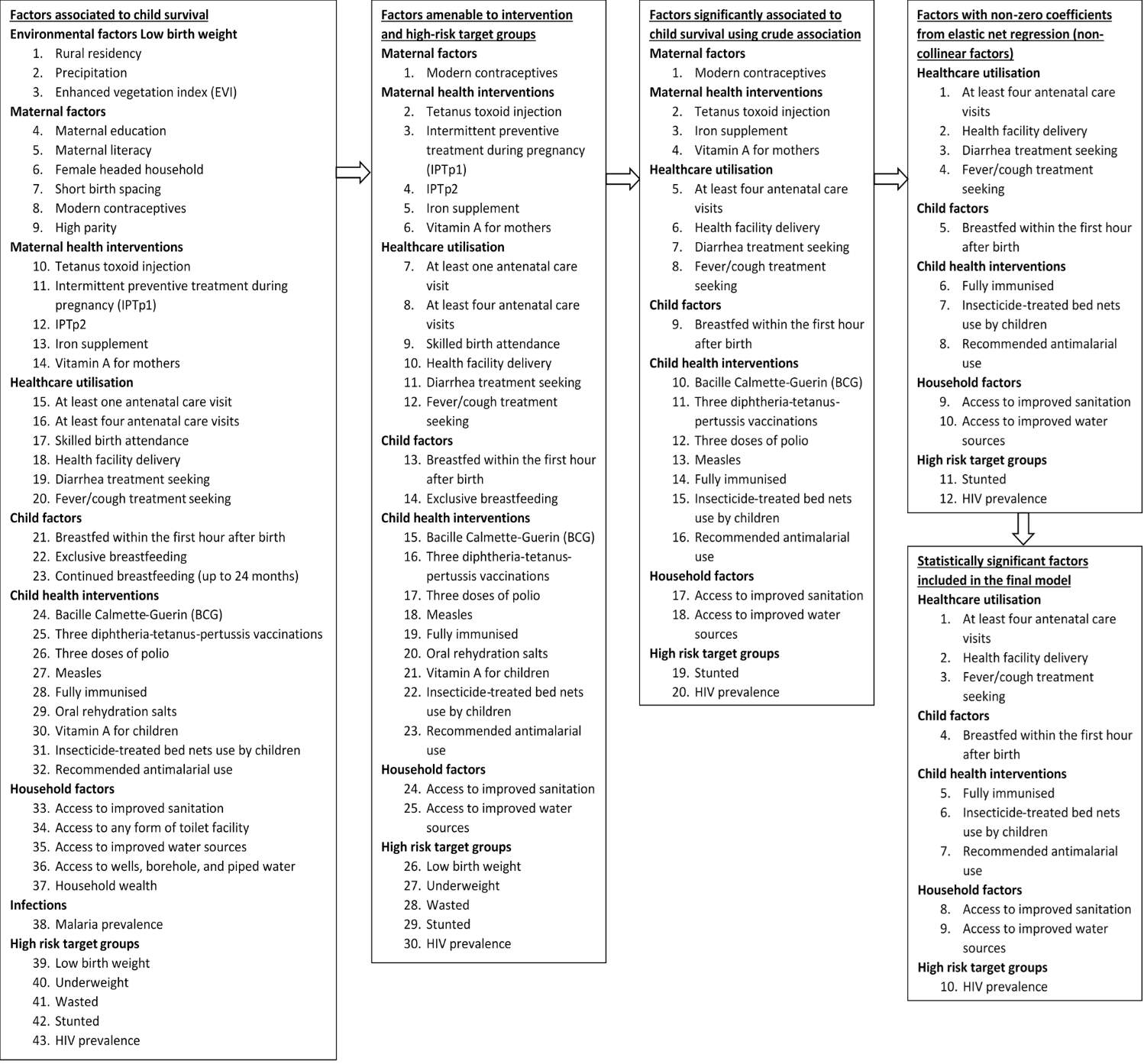


To validate contextual associations in Kenya, bivariate log-linear regressions were fitted to explore the crude association of child survival factors amenable to intervention and U5M based on historical trends observed between (2003-2014) (Table 1). Among the covariates with significant effects (p<0.2) from the bivariate analysis, factors whose contribution was captured by other variables under consideration were excluded to reduce circularity and confounding. We excluded DTP3, Polio3, measles and BCG vaccines whose effects were captured by fully immunised status. To select the most predictive factors from the remaining factors while accounting for collinearity we fit two ENR models assessing collinearity between antenatal visits (ANC) and interventions provided during ANC (Table 2).

**Table B:** **Determinants of child mortality amenable to interventions and** **crude association with under-five mortality for the period 2003-2014**

| **Indicators** | **Coefficient (95% CI)** | **pvalue** | **Adjusted R^2^** |
| --- | --- | --- | --- |
| **Maternal interventions** | | | |
| Contraceptive use | -0.6344 (-0.6992--0.5557) | <0.0001 | 0.0895 |
| Vitamin A (mother) | -0.4182 (-0.4853--0.3424) | <0.0001 | 0.0671 |
| **Pregnancy-related interventions** | | | |
| At least 1 antenatal visit (ANC1) | -0.1401 (-0.3388-0.1183) | 0.26 | 0.0003 |
| At least 4 antenatal visits (ANC4) | 0.9428 (0.5169-1.4882) | <0.0001 | 0.0252 |
| Iron supplement | -0.2477 (-0.3063--0.1841) | <0.0001 | 0.0429 |
| IPTP1 | 1.0739 (-0.7625-1.5126) | 0.682 | -0.0032 |
| IPTP2 | 1.2642 (-0.76832-2.08019) | 0.355 | -0.0005 |
| At least 2 tetanus injections | -0.7996 (-0.8672--0.6976) | <0.0001 | 0.0529 |
| Health facility delivery | -0.4749 (-0.5416--0.3985) | <0.0001 | 0.0765 |
| Skilled birth attendance | - 0.2023 (-0.3179-0.0064) | 0.3432 | 0.0124 |
| **Child health care seeking and preventative interventions** | | | |
| Fully immunized | -0.5604 (-0.6256--0.4839) | <0.0001 | 0.0883 |
| DPT3 vaccine | -0.5826 (-0.6534--0.4974) | <0.0001 | 0.0753 |
| Polio vaccine | -0.5824 (-0.6600--0.4870) | <0.0001 | 0.0621 |
| Measles vaccine | -0.8645 (-0.8924--0.8293) | <0.0001 | 0.2178 |
| Exclusive breastfeeding | 0.0363 (-0.1913-0.3281) | 0.778 | -0.0009 |
| Breastfeeding within the first hour | -0.8587 (-0.8806--0.8329) | <0.0001 | 0.3353 |
| Diarrhea treatment | -0.4988 (-0.6530--0.2761) | <0.0001 | 0.012 |
| Fever treatment | -0.9472 (-0.9628--0.9253) | <0.0001 | 0.2102 |
| Antimalarial use | -14.9407 (-24.1205- -9.1155) | <0.0001 | 0.1677 |
| Child ITN use  Vitamin A for children  Oral rehydration salts | -0.1939 (-0.2734--0.1057)  -0.1143 (-0.3646-0.2346)  0.2152 (-0.2594-0.9937) | <0.0001  0.473  0.440 | 0.0149  0.0009  0.0007 |
| **Child health indicators** | | | |
| Stunting | 1.0758(0.0662-3.0413) | 0.032 | 0.064 |
| Wasting | -1.3426(-1.9866- -0.69853) | <0.0001 | 0.0272 |
| Underweight | -1.1117 (-1.5863-0.06371) | 0.524 | 0.0346 |
| Low birth weight (<2500g) | 0.6194 (-0.8972-24.5348) | 0.732 | -0.0009 |
| **Water, Sanitation, and Hygiene Indicators (WASH)** | | | |
| Better water access (piped water) | -0.3144 (-0.4005--0.2158) | <0.0001 | 0.0277 |
| Better sanitation (flush toilets) | -0.2231 (-0.3051--0.1315) | <0.0001 | 0.0178 |
| **Community disease prevalence** | | | |
| HIV prevalence | 5.79299 (5.11690- 6.46907) | <0.0001 | 0.3339 |

**Table C:** **Alternate Elastic Net Regression results for variable selection**

| **Model** | **MSE** | **Factors with zero coefficients (excluded)** |
| --- | --- | --- |
| Model 1; Antenatal care visits (ANC4), Iron, tetanus injection and Vitamin A supplements | 0.3759 | Diarrhea treatment, contraceptive use and Tetanus injection |
| Model 2; Antenatal care visits (ANC4) and PCA index* | 0.34211 | Diarrhea treatment, contraceptive use and PCA index |
| Model 3; Base model | 0.18427 | Diarrhea treatment, contraceptive use, Tetanus injection, Vitamin A and iron supplements |

**Calculating annual rate of change (ARC) for intervention coverage**

To obtain the ARC for each intervention coverage from the predefined 2022 or 2025 targets and the baseline coverage (2014 estimates) we used the equation below:

$\boldsymbol{Annual rate of change(\%)}\boldsymbol{=}\frac{\boldsymbol{((target coverage-baseline)/(baseline))}}{\boldsymbol{t}}\boldsymbol{*100}$

where t is either 8 for 2022 targets or 11 for 2025 targets

**Assumptions on U5M projections**

To assess the impact of the four alternate intervention scale-up scenarios namely; Scenario 1; 2014 Best performing county coverage, scenario 2; Highest annual rate of change 2003-2014, scenario 3; 2022 and 2025 National projected coverage and scenario 4; 2022 and 2025 national health strategic plans targets, we made several assumptions about interventions and how their associations with U5M were modelled.

1. We assume that historical trends in associations between interventions and U5M from the observed data (2003-2014) would still apply over the study period between 2015-2025 (projection period).
2. The changes in U5M were in response to changes intervention coverage and the impact of confounders such as distal factors (for example socioeconomic status) are mediated by changes in interventions coverage. This assumption suggests causal effects of the interventions adopted in the analysis based on prior research identifying these factors as drivers of U5M in Kenyan context
3. We used linear interpolation to estimate the impact of various interventions. Therefore, we assume that the impact of increasing intervention coverage or reduction of disease prevalence would be equivalent regardless of the initial values.
4. We assume that intervention scale-up independently and additively affect U5M. Therefore, we do explicitly account for the interactions between changes in intervention coverage for related interventions such as access to clean water and improved sanitation.

**References**

1.Macharia PM, Joseph NK, Snow RW, Sartorius B, Okiro EA. The impact of child health interventions and risk factors on child survival in Kenya, 1993-2014: a Bayesian spatio-temporal analysis with counterfactual scenarios. BMC Med. 2021;19(1):102.
