# Supplementary material for: Progress towards achieving child survival goals in Kenya after devolution: Geospatial analysis with scenario-based projections, 2015–2025
Source: PLOS Glob Public Health. 2022 Oct 5;2(10):e0000686. doi: 10.1371/journal.pgph.0000686 (PMC10021401; doi:10.1371/journal.pgph.0000686)
Supplement: S2 File — (DOCX) [file pgph.0000686.s002.docx]

| **County** | **U5M** | **Antenatal care (4 visits)** | **Fully immunised** | **Better sanitation** | **Early breastfeeding** | **Fever treatment** | **Health facility delivery** | **Access to improved water** | **Antimalarial use** | **Child ITN use** | **HIV prevalence** |
| --- | --- | --- | --- | --- | --- | --- | --- | --- | --- | --- | --- |
| Baringo | -1.26 | 0.1 | 1.66 | 1.61 | 1.19 | 1 | 5.29 | 4.99 | 4.19 | 19.57 | -5.47 |
| Bomet | -0.49 | -0.55 | 2.08 | 0.6 | 1.96 | 1.51 | 4.62 | 1.22 | 4.99 | 27.84 | -5.47 |
| Bungoma | -2.39 | -0.98 | 4.85 | 0.14 | 5.54 | 3.18 | 5.72 | 1.29 | 8.44 | 20.4 | -5.18 |
| Busia | -2.97 | 0.23 | 3.22 | 0.24 | 4.77 | 3 | 6.64 | 1.55 | 8.5 | 20.88 | -5.18 |
| Embu | 2.22 | 0.87 | 1.45 | 0.17 | 0.09 | 0.17 | 1.69 | 2.1 | -0.31 | 18.72 | -4.43 |
| Garissa | -1.13 | 3.12 | 9.76 | 0.95 | 2.42 | 0.17 | 3.7 | 3.71 | -4.91 | 23.5 | -9.12 |
| Homa Bay | -3.43 | 1.8 | 7.97 | 3.25 | 3.2 | 2.55 | 7.57 | 1.9 | 8.51 | 22.05 | -2.35 |
| Isiolo | -1.49 | 3.09 | 4.05 | 4.39 | 1.18 | 0.61 | 3.45 | 2.91 | 1.96 | 24.17 | -4.43 |
| Kajiado | -0.9 | 0.85 | 3.26 | 1.35 | 0.54 | 1.48 | 4.55 | 0.65 | -2.94 | 22.99 | -5.47 |
| Kakamega | -3.19 | 0.1 | 4.27 | 0.14 | 4.41 | 3.18 | 5.76 | 1.53 | 9.61 | 21.16 | -5.18 |
| Elgeyo-Marakwet | -1.67 | -0.14 | 1.93 | 2.26 | 1.92 | 1.88 | 3.23 | 3.88 | 5.14 | 24.17 | -5.47 |
| Kericho | -1.48 | 0.71 | 1.82 | 1.09 | 2.04 | 1.79 | 4.98 | 2.34 | 4.52 | 22.99 | -5.47 |
| Kiambu | 0.49 | 0.9 | 1.84 | 0.02 | -0.15 | 1.5 | 2.39 | 1.4 | -1 | 21.55 | -3.75 |
| Kilifi | -1.2 | 2.37 | 2.63 | 3.57 | 10.83 | 1.46 | 8.1 | 0.87 | -10.35 | 22.7 | -4.36 |
| Kirinyaga | 1.93 | 0.11 | 1.16 | 0.01 | -0.2 | 0.24 | 1.88 | 2.43 | -2.06 | 16.4 | -3.75 |
| Kisii | -3.02 | 1.32 | 4.63 | 0.12 | 1.1 | 2.14 | 4.95 | -0.27 | 4.45 | 20.02 | -2.35 |
| Kisumu | -4.12 | 1.56 | 4.69 | 0.68 | 3.3 | 2.1 | 5.23 | 0.72 | 13.23 | 19.45 | -2.35 |
| Kitui | -1.57 | 2.22 | 2.85 | 2.45 | 0.99 | 0.56 | 5.17 | 5.7 | -3.35 | 23.8 | -4.43 |
| Kwale | -1.12 | 1.31 | 1.3 | 6.27 | 11.16 | 1.65 | 8.56 | 0.64 | -0.68 | 21.34 | -4.36 |
| Laikipia | 0 | 0.58 | 2.11 | -0.28 | 1.38 | 0.6 | 4.22 | 3.29 | 3.51 | 20.21 | -5.47 |
| Lamu | 1.55 | 2.58 | 3.52 | 0.78 | 3.56 | 0.53 | 0.76 | 2.3 | -11.09 | 15.31 | -4.36 |
| Machakos | -0.32 | 1.16 | 2.19 | 0.38 | -0.24 | 0.8 | 5.56 | 2.27 | -1.7 | 23.83 | -4.43 |
| Makueni | -1.22 | 2.33 | 2.24 | 0.43 | 1.17 | 1.36 | 6.13 | 2.89 | -1.94 | 28.12 | -4.43 |
| Mandera | -4.33 | 5.17 | 5.59 | 4.11 | 3.57 | 4.27 | 8.1 | 4.39 | 4.13 | 34.36 | -9.12 |
| Marsabit | -1.57 | 4.97 | 5.76 | 2.25 | 0.64 | 1.07 | 4.95 | 2.23 | 0.7 | 16.1 | -4.43 |
| Meru | 2.17 | 1.3 | 3.28 | 0.17 | 0.47 | 0.21 | 2.3 | 2.09 | -2.07 | 20.12 | -4.43 |
| Migori | -2.99 | 1.6 | 7.76 | 2.14 | 2.06 | 2.81 | 6.42 | 0.26 | 6.33 | 21.27 | -2.35 |
| Mombasa | 0.13 | 1.82 | 2.69 | 0.23 | 7.78 | 2.16 | 3.88 | -0.38 | -1.92 | 16.03 | -4.36 |
| Murang'a | 1.08 | 0.49 | 1.4 | 0.04 | 1.06 | 0.95 | 2.86 | 3.54 | -7.44 | 21.48 | -3.75 |
| Nairobi | 1.44 | 0.67 | 2.69 | 0.11 | 0.99 | 1.03 | 1.41 | 0.08 | 2.22 | 19.26 | -2.38 |
| Nakuru | 1.49 | 0.34 | 2.36 | 0.09 | 0.77 | 1.97 | 3.86 | 1.45 | 7.28 | 25.17 | -5.47 |
| Nandi | -2.87 | 0.94 | 2.79 | 0.61 | 2.88 | 1.76 | 6.08 | 1.42 | 6.28 | 27.82 | -5.47 |
| Narok | -1.03 | 0.84 | 5.12 | 4.41 | 0.25 | 2.13 | 7.09 | 2.03 | 8.16 | 26.24 | -5.47 |
| Nyamira | -1.51 | 0.7 | 3.64 | 0.03 | 2.37 | 1.61 | 4.16 | 2.09 | 0.72 | 18.93 | -2.35 |
| Nyandarua | 2.92 | 0.25 | 2.12 | 0 | 0.59 | 1.06 | 3.26 | 2.29 | 0.6 | 23.89 | -3.75 |
| Nyeri | 1.89 | 0.48 | 1.62 | 0.01 | 0.77 | 0.42 | 1.3 | 2 | -5.06 | 22.92 | -3.75 |
| Samburu | -2.07 | 2.59 | 3.26 | 5.05 | 0.66 | 0.61 | 3.51 | 3.85 | 1.45 | 17.19 | -5.47 |
| Siaya | -4.22 | 1.1 | 5.59 | 1.12 | 3.92 | 3.25 | 5.88 | 1.78 | 11.58 | 21.96 | -2.35 |
| Taita Taveta | -1.32 | 2.07 | 0.78 | 0.05 | 2.74 | 0.54 | 4.81 | 2.18 | -5.8 | 26.93 | -4.36 |
| Tana River | -1.06 | 3.49 | 3.29 | 4.75 | 3.63 | 0.62 | 3.66 | 5.16 | -2.44 | 20.98 | -4.36 |
| Tharaka | 0.14 | 1.53 | 2.37 | 0.21 | 0.12 | 0.33 | 2.33 | 1.85 | -1.9 | 17.78 | -4.43 |
| Trans Nzoia | -0.82 | -0.47 | 3.37 | 0.42 | 3.46 | 2.49 | 5.63 | 2.01 | 7.33 | 19.42 | -5.47 |
| Turkana | -2.06 | 1.86 | 4.35 | 13.03 | 1.72 | 1.32 | 13.84 | 2.78 | 7.27 | 24.5 | -5.47 |
| Uasin Gishu | -1.79 | 0.83 | 2.99 | 0.28 | 2.6 | 2.25 | 4.56 | 0.92 | 4.79 | 22.41 | -5.47 |
| Vihiga | -2.32 | 1.18 | 3.68 | 0 | 3.64 | 3.01 | 7.64 | -0.45 | 11.32 | 23.91 | -5.18 |
| Wajir | -2.61 | 5.3 | 7.84 | 3.87 | 3.47 | 2.71 | 7.65 | 3.61 | -6.76 | 29.23 | -9.12 |
| West Pokot | -2.42 | -0.96 | 3.02 | 9.17 | 0.79 | 1.79 | 5.63 | 7.77 | 2.56 | 20.9 | -5.47 |
| **Average** | **-1.44 (-1.61- -0.54)** | **1.36(0.94-1.78)** | **3.47(2.89-4.04)** | **1.76(0.99-2.53)** | **2.41(1.68-3.13)** | **1.57(1.28-1.80)** | **4.91(4.22-5.61)** | **2.24(1.76-2.72)** | **1.83(0.13-3.53)** | **22.13(21.02-23.23)** | **-4.71 (-5.17- -4.25)** |

**Table A: County-specific annualised rates of change (ARC) for the 10 selected high impact factors and U5M based on the period between 2003-2014**
