# Supplementary material for: Progress towards achieving child survival goals in Kenya after devolution: Geospatial analysis with scenario-based projections, 2015–2025
Source: PLOS Glob Public Health. 2022 Oct 5;2(10):e0000686. doi: 10.1371/journal.pgph.0000686 (PMC10021401; doi:10.1371/journal.pgph.0000686)
Supplement: S3 File — (DOCX) [file pgph.0000686.s003.docx]

**Fig A: Trace plots history for model parameters based on chain 1(red) and chain 2(blue).** The two chains appear to be overlapping indicating, there is some reasonable confidence that convergence and or stabilization was achieved.
