# Supplementary material for: Progress towards achieving child survival goals in Kenya after devolution: Geospatial analysis with scenario-based projections, 2015–2025
Source: PLOS Glob Public Health. 2022 Oct 5;2(10):e0000686. doi: 10.1371/journal.pgph.0000686 (PMC10021401; doi:10.1371/journal.pgph.0000686)
Supplement: S4 File — (PPTX) [file pgph.0000686.s004.pptx]

## Slide 1
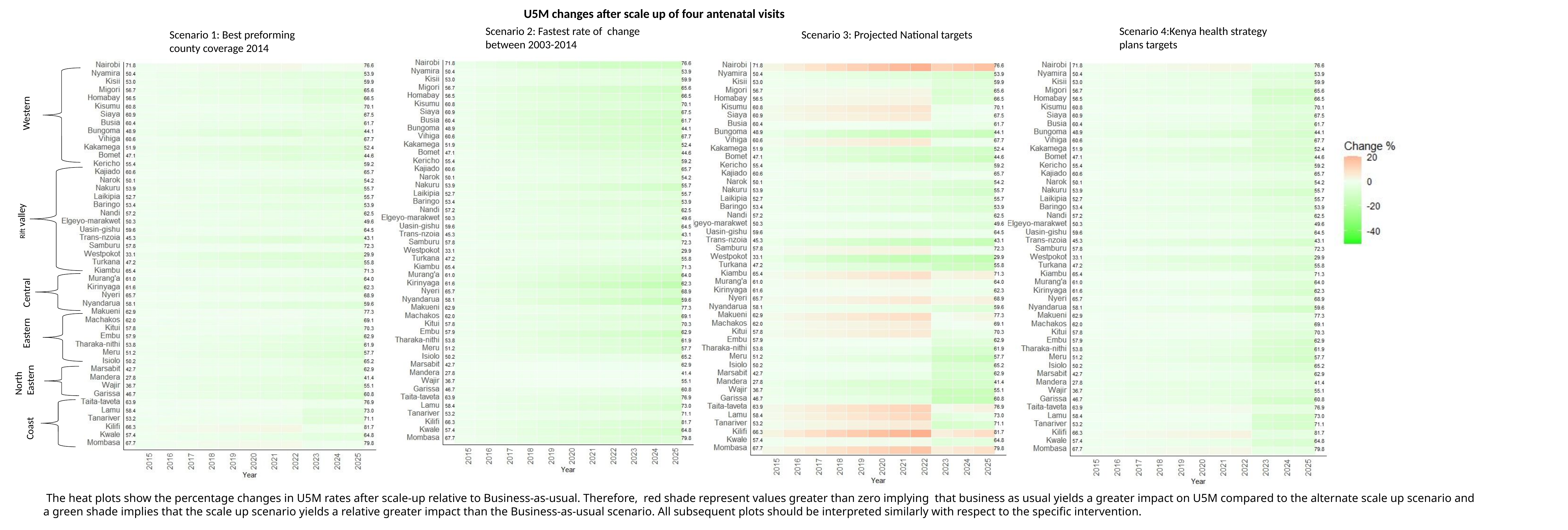

U5M changes after scale up of four antenatal visits
Scenario 2: Fastest rate of change between 2003-2014
Scenario 4:Kenya health strategy plans targets
Scenario 3: Projected National targets
Scenario 1: Best preforming county coverage 2014
Western
Rift valley
Central
Eastern
North
Eastern
Coast
 The heat plots show the percentage changes in U5M rates after scale-up relative to Business-as-usual. Therefore, red shade represent values greater than zero implying that business as usual yields a greater impact on U5M compared to the alternate scale up scenario and a green shade implies that the scale up scenario yields a relative greater impact than the Business-as-usual scenario. All subsequent plots should be interpreted similarly with respect to the specific intervention.

## Slide 2
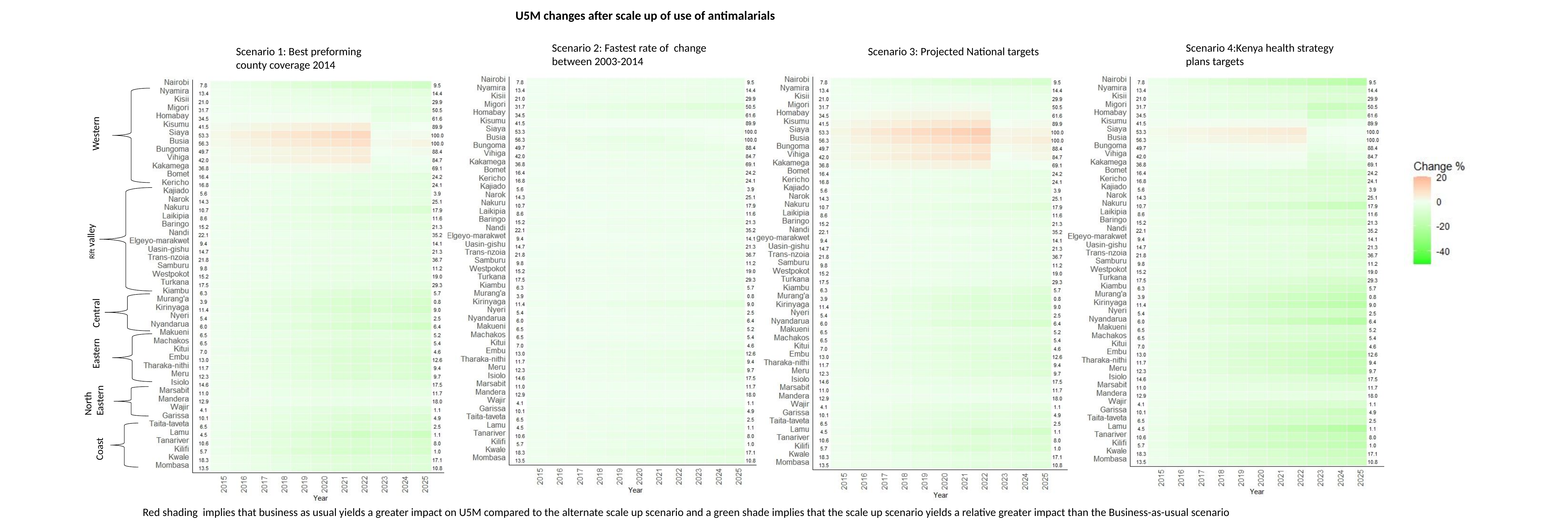

U5M changes after scale up of use of antimalarials
Scenario 2: Fastest rate of change between 2003-2014
Scenario 4:Kenya health strategy plans targets
Scenario 3: Projected National targets
Scenario 1: Best preforming county coverage 2014
Western
Rift valley
Central
Eastern
North
Eastern
Coast
Red shading implies that business as usual yields a greater impact on U5M compared to the alternate scale up scenario and a green shade implies that the scale up scenario yields a relative greater impact than the Business-as-usual scenario

## Slide 3
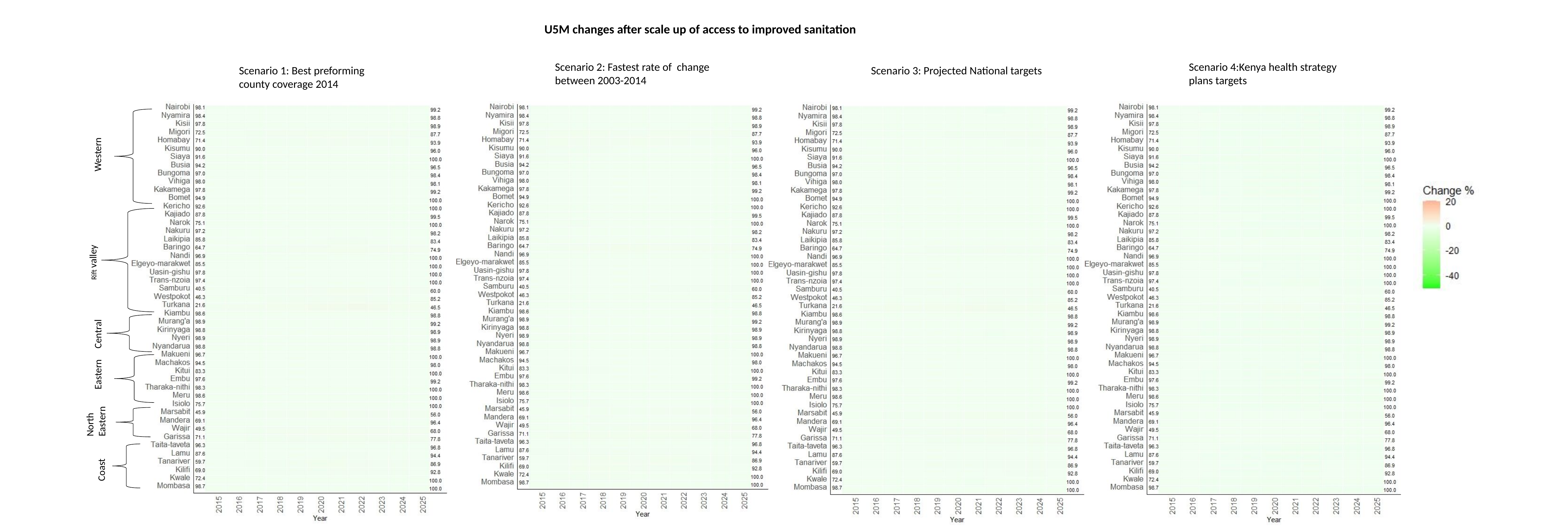

U5M changes after scale up of access to improved sanitation
Scenario 2: Fastest rate of change between 2003-2014
Scenario 4:Kenya health strategy plans targets
Scenario 3: Projected National targets
Scenario 1: Best preforming county coverage 2014
Western
Rift valley
Central
Eastern
North
Eastern
Coast

## Slide 4
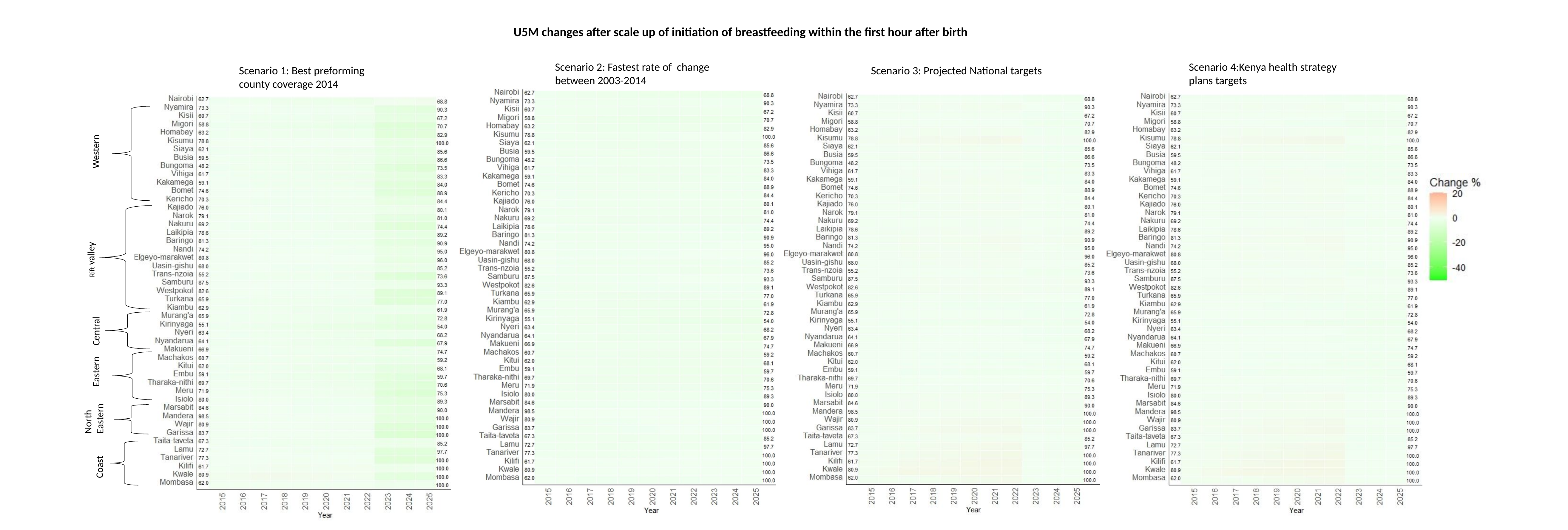

U5M changes after scale up of initiation of breastfeeding within the first hour after birth
Scenario 2: Fastest rate of change between 2003-2014
Scenario 4:Kenya health strategy plans targets
Scenario 3: Projected National targets
Scenario 1: Best preforming county coverage 2014
Western
Rift valley
Central
Eastern
North
Eastern
Coast

## Slide 5
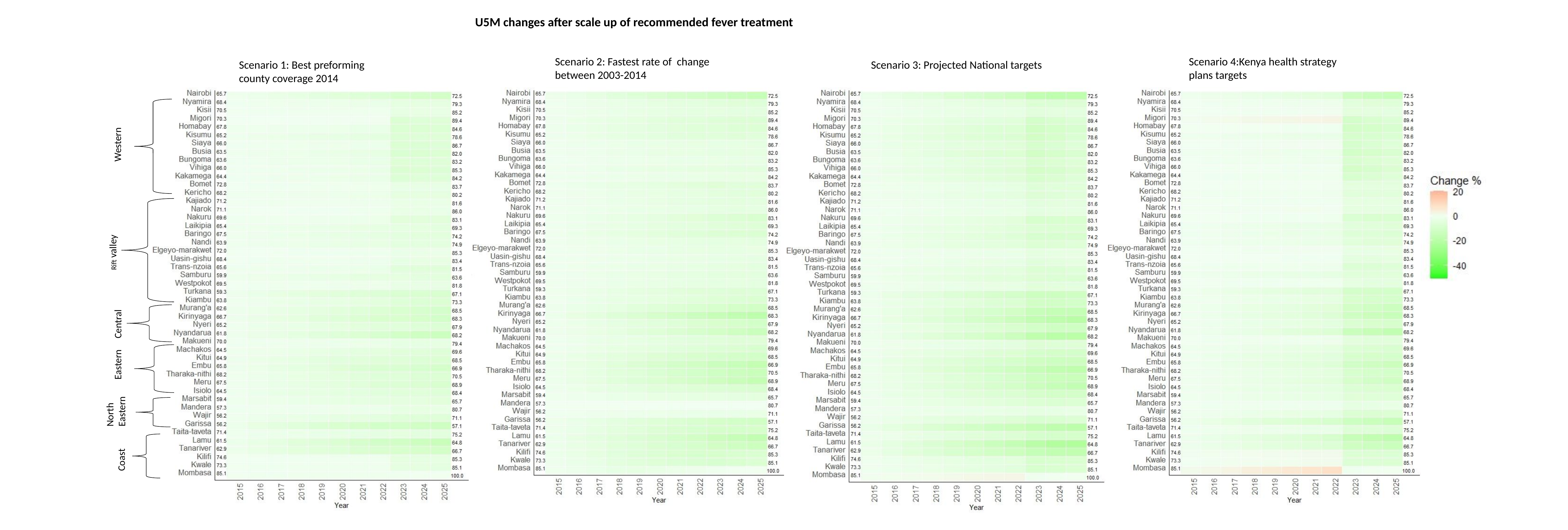

U5M changes after scale up of recommended fever treatment
Scenario 2: Fastest rate of change between 2003-2014
Scenario 4:Kenya health strategy plans targets
Scenario 3: Projected National targets
Scenario 1: Best preforming county coverage 2014
Western
Rift valley
Central
Eastern
North
Eastern
Coast

## Slide 6
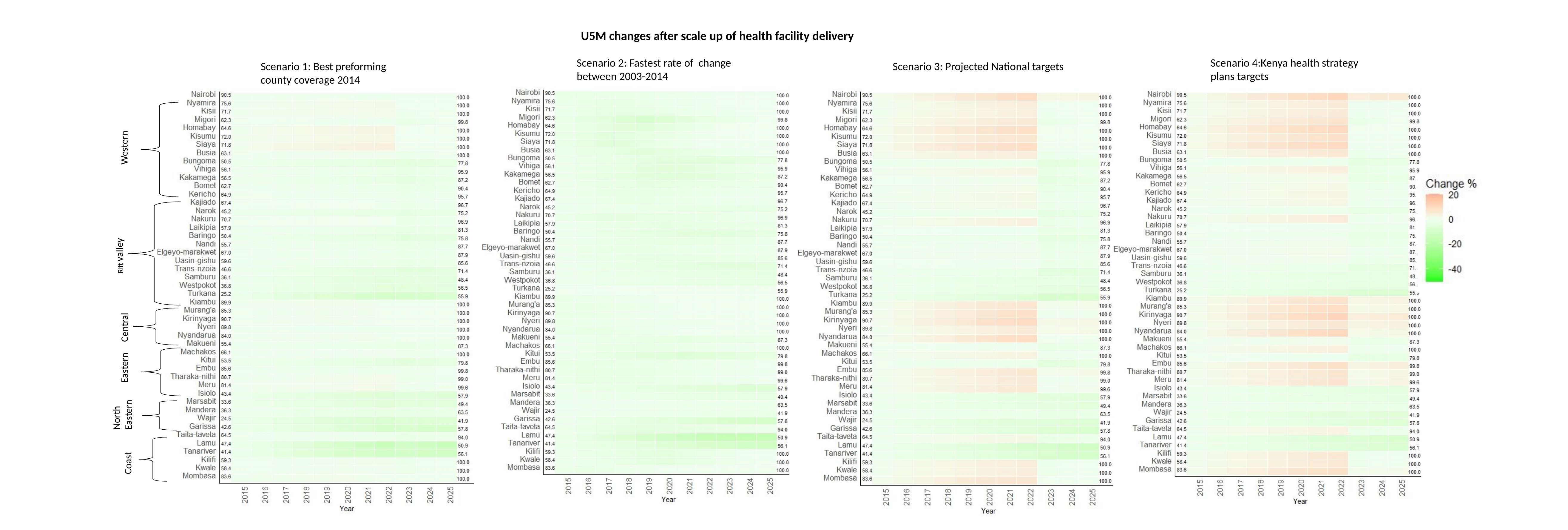

U5M changes after scale up of health facility delivery
Scenario 2: Fastest rate of change between 2003-2014
Scenario 4:Kenya health strategy plans targets
Scenario 3: Projected National targets
Scenario 1: Best preforming county coverage 2014
Western
Rift valley
Central
Eastern
North
Eastern
Coast

## Slide 7
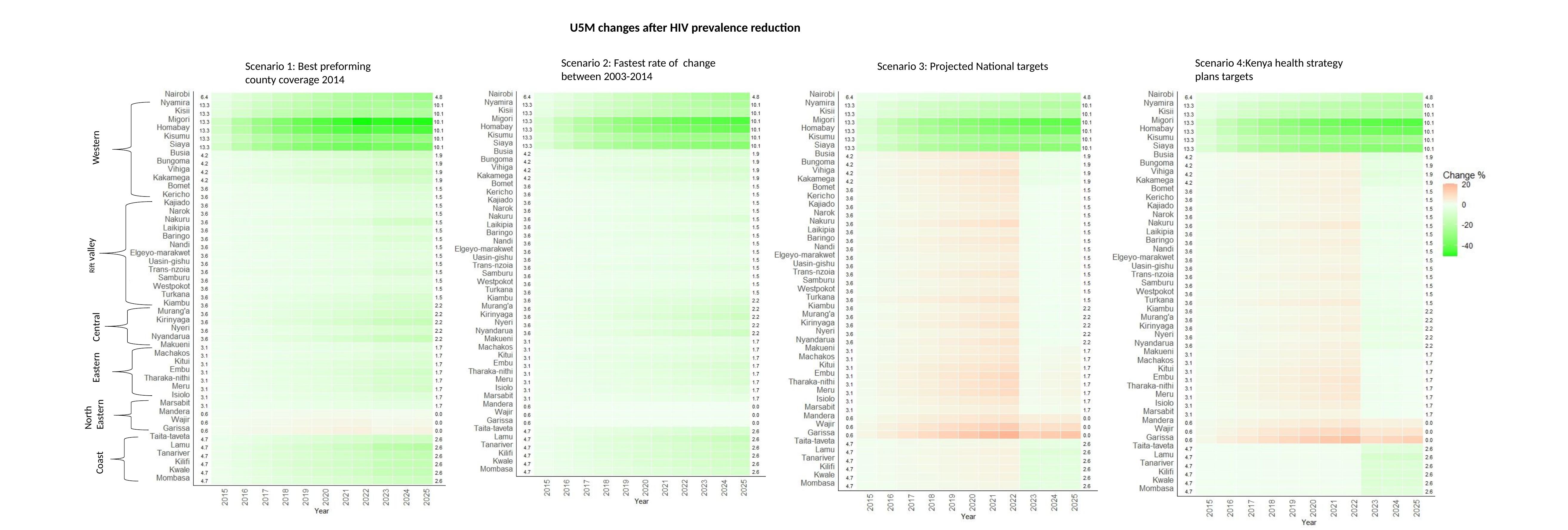

U5M changes after HIV prevalence reduction
Scenario 2: Fastest rate of change between 2003-2014
Scenario 4:Kenya health strategy plans targets
Scenario 3: Projected National targets
Scenario 1: Best preforming county coverage 2014
Western
Rift valley
Central
Eastern
North
Eastern
Coast

## Slide 8
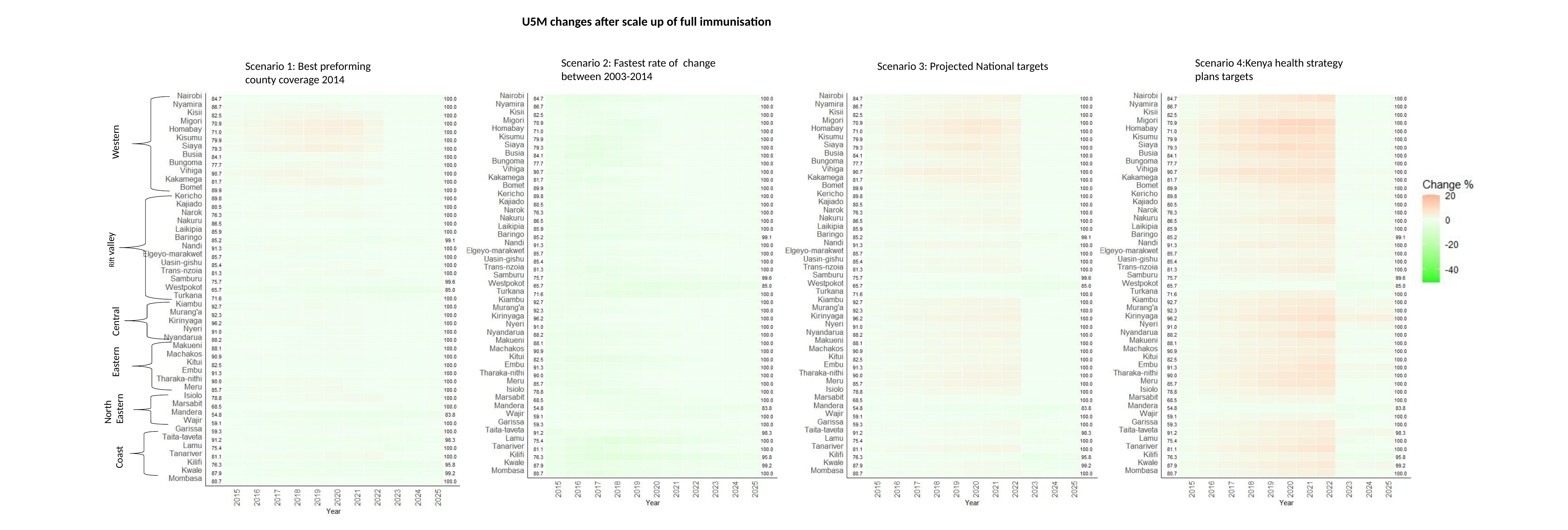

U5M changes after scale up of full immunisation
Scenario 2: Fastest rate of change between 2003-2014
Scenario 4:Kenya health strategy plans targets
Scenario 3: Projected National targets
Scenario 1: Best preforming county coverage 2014
Western
Rift valley
Central
Eastern
North
Eastern
Coast

## Slide 9
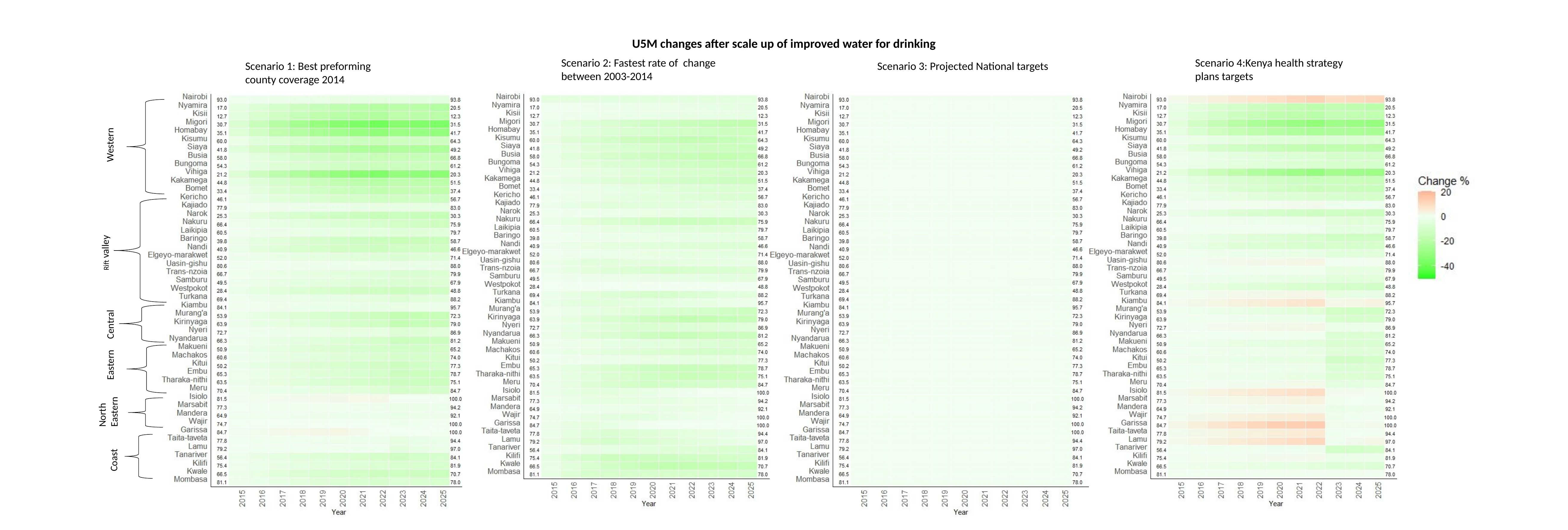

U5M changes after scale up of improved water for drinking
Scenario 2: Fastest rate of change between 2003-2014
Scenario 4:Kenya health strategy plans targets
Scenario 3: Projected National targets
Scenario 1: Best preforming county coverage 2014
Western
Rift valley
Central
Eastern
North
Eastern
Coast

## Slide 10
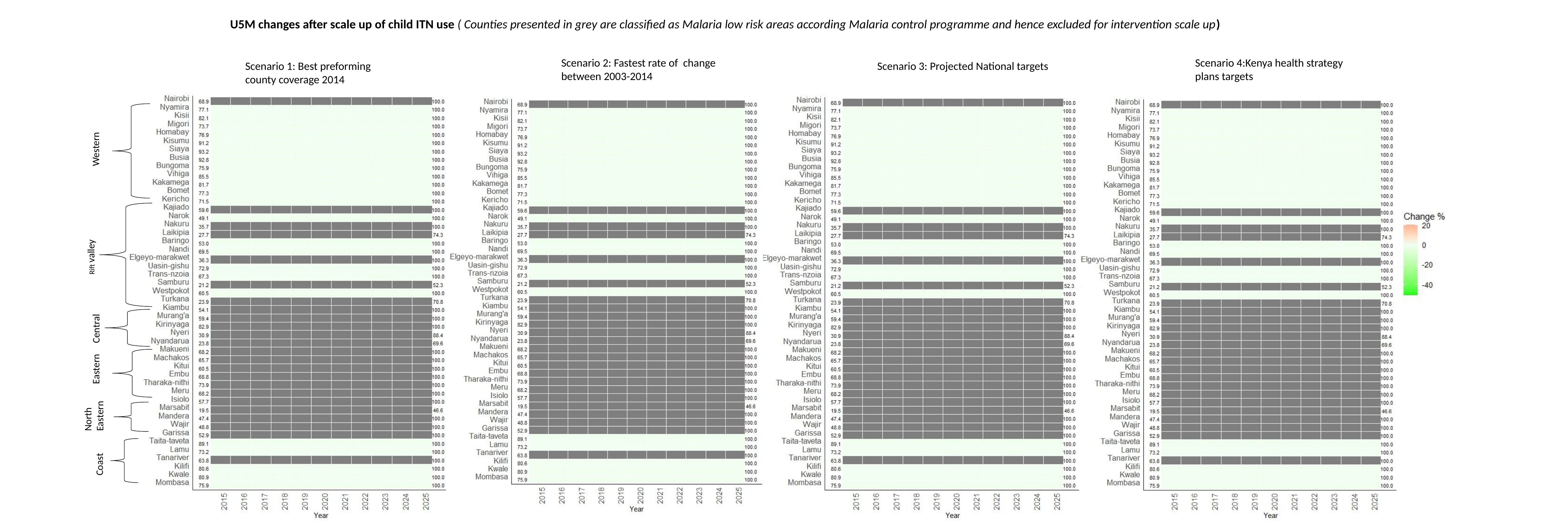

U5M changes after scale up of child ITN use ( Counties presented in grey are classified as Malaria low risk areas according Malaria control programme and hence excluded for intervention scale up)
Scenario 2: Fastest rate of change between 2003-2014
Scenario 4:Kenya health strategy plans targets
Scenario 3: Projected National targets
Scenario 1: Best preforming county coverage 2014
Western
Rift valley
Central
Eastern
North
Eastern
Coast
